# Nanomaterial-Based Repurposing of Macrophage Metabolism and Its Applications

**DOI:** 10.1007/s40820-024-01455-9

**Published:** 2024-07-15

**Authors:** Tingting Meng, Danfeng He, Zhuolei Han, Rong Shi, Yuhan Wang, Bibo Ren, Cheng Zhang, Zhengwei Mao, Gaoxing Luo, Jun Deng

**Affiliations:** 1 Institute of Burn Research, Southwest Hospital, State Key Laboratory of Trauma and Chemical Poisoning, Army Medical University, Chongqing, 400038 People’s Republic of China; 2 Department of Breast Surgery, Gansu Provincial Hospital, Lanzhou, Gansu 730030 People’s Republic of China; 3 MOE Key Laboratory of Macromolecular Synthesis and Functionalization, Department of Polymer Science and Engineering, Zhejiang University, Hangzhou, 310027 People’s Republic of China

**Keywords:** Immunomodulatory nanomaterial, Macrophage polarization, Macrophage metabolic reprogramming, Immune engineering

## Abstract

Macrophage immunotherapy represents an emerging therapeutic approach aimed at modulating the immune response to alleviate disease symptoms. Nanomaterials (NMs) have been engineered to monitor macrophage metabolism, enabling the evaluation of disease progression and the replication of intricate physiological signal patterns. They achieve this either directly or by delivering regulatory signals, thereby mapping phenotype to effector functions through metabolic repurposing to customize macrophage fate for therapy. However, a comprehensive summary regarding NM-mediated macrophage visualization and coordinated metabolic rewiring to maintain phenotypic equilibrium is currently lacking. This review aims to address this gap by outlining recent advancements in NM-based metabolic immunotherapy. We initially explore the relationship between metabolism, polarization, and disease, before delving into recent NM innovations that visualize macrophage activity to elucidate disease onset and fine-tune its fate through metabolic remodeling for macrophage-centered immunotherapy. Finally, we discuss the prospects and challenges of NM-mediated metabolic immunotherapy, aiming to accelerate clinical translation. We anticipate that this review will serve as a valuable reference for researchers seeking to leverage novel metabolic intervention-matched immunomodulators in macrophages or other fields of immune engineering.

## Highlights

- A large-scale metabolic adaptation in M1-polarized macrophages compared with M2 macrophages, along with multiple altered metabolite parameters that can serve as potential targets, have been discussed.
- Nanomaterial-based repurposing of macrophage metabolism and its applications are systematically summarized and illustrated using representative examples.
- Potential challenges, additional information about future research objectives, and priorities of nanomaterial-based macrophage immunotherapy are highlighted.

## Graphical abstract

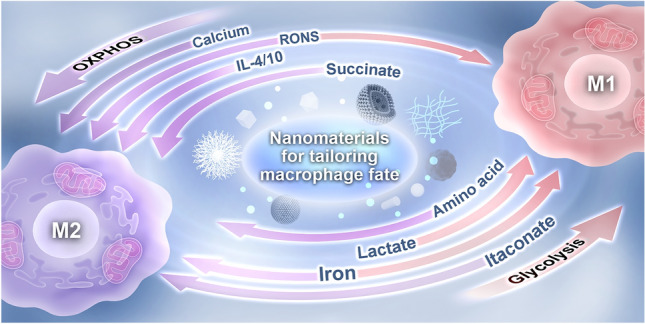

## Introduction

Macrophages are vital cellular components of the innate immune system, playing crucial roles in regulating inflammatory responses to maintain tissue homeostasis [1–3]. However, macrophage responses represent a double-edged sword, acting both as a protective “safety guard” and as a potential instigator of disease [4]. They exhibit a full spectrum of polarized phenotypes, encompassing proinflammatory-like (M1) and anti-inflammatory-like (M2) states [5–9] (Table 1). Macrophage polarization (M1/M2) remains dynamic and finely balanced due to their heterogeneity and plasticity, adapting to tailored cues in the physiological microenvironment [9, 10].

**Table 1:** Potential metabolic targets of macrophages and M1/M2-like biomarkers

Metabolic targets	M1 biomarkers	M2 biomarkers
(1) RONS	(1) Activated inducer: LPS, IFN-γ	(1) Activated inducer: IL-4, IL-13
Itaconate	(2) Surface marker: CD80, CD86, CD68, TLR-2/4	(2) Surface marker: CD163, CD206
Iron	(3) Secreted cytokines: TNF-α, IL-1β, IL-6/12/23/17A, IFN-β	(3) Secreted cytokines: IL-10, TGF-β, VEGFα, bFGF
IL-4	(4) Secreted chemokines: CCL-2/9/11, CXCL-2, CCR7	(4) Secreted chemokines: CCL-1/17/
IL-10	(5) Marker gene: *NOS2*, *Nfkbiz*, *IFN-α*, *S100a9a, IFN-α/β*, *Edn-*, *Irf-5*	(5) Marker gene: *Arg-1*, *Fizz-1*, *Ym-1*, *KLF-4, Smad3*
Calcium	(6) Signaling molecule: iNOS, Socs-3, STAT-1	(6) Signaling molecule: STAT6, Arg-1
Amino acid		(7) Receptor: MerTK, TLR1/8
Lactate		

Immunometabolism has provided novel insights into the hyperactivation of macrophage metabolism. M1 macrophages rewrite their metabolic program to coordinate the adaptive immune response compared to M2 macrophages, which rely on oxidative phosphorylation (OXPHOS). The metabolic adaption includes an interrupted tricarboxylic acid (TCA) cycle, increased activation of glycolysis and the pentose phosphate pathway (PPP), and alterations in various metabolic parameters such as succinate or itaconate, reactive oxygen and nitrogen species (RONS), and amino acids [11–15]. Several excellent reviews have elaborately described the intricate relationship between macrophage polarization and metabolism [11, 13, 14, 16]. This alteration-coordinated metabolic rewiring profoundly influences macrophage gene expression, leading to unbalanced M1/M2 polarization [16–19], which determines the progression of diseases such as osteoarthritis (OA) [20], acute lung injury (ALI) [21], myocardial infarction (MI) [22], diabetic wounds [23, 24], atherosclerosis [25], and inflammatory bowel disease (IBD) [26]. Conversely, tumor-associated macrophages (TAMs) initially exhibit a higher glycolysis rate to support antitumor M1 activity, but gradually transition toward more efficient OXPHOS and fatty acid oxidation (FAO) to promote protumor M2 functions, thereby facilitating tumor immune escape [18, 27–30]. Given the critical role of altered metabolic profiles as positive or negative regulators of macrophage fate in driving disease evolution, in situ tracking of macrophage metabolism holds significant importance for elucidating disease progression. Moreover, intervening in metabolism to exacerbate metabolic imbalance or restore metabolic homeostasis has been exploited as a fascinating avenue to reshape macrophage phenotypes and elicit the corresponding immune response against diseases.

Nanomaterials (NMs) have been extensively fabricated to display and transmit macrophage regulatory signals in a precise and closely physiological manner, simulating artificial microenvironments and guiding macrophage fate [31]. This is benefited by their intrinsic physicochemical properties and macrophage phagocytosis, in which foreign particles are preferentially internalized [32, 33]. Recently, the metabolic pathways of M1/M2 macrophages have garnered increasing attention from researchers, leading to their adoption in macrophage diagnosis and therapeutic exploitation. This trend has facilitated the blossom of suitable theranostic agents for macrophage-driven diseases. Several researchers have exploited imaging probes, integrating them with advanced technologies such as magnetic resonance imaging (MRI), to track macrophage activity in situ and reveal disease progression [34–38], such as dual-responsive nanoreporters for monitoring macrophage phenotypes [39]. NMs serve as emerging immunomodulatory weapons, enabling the remote manipulation of macrophage metabolism [19, 31, 40], thereby directing their polarization toward desired phenotypes for disease therapy [41], such as nanozymes combating inflammatory diseases by ROS scavenging-primed M2-like [42, 43]. However, there remains a lack of in-depth summary and discussion regarding current strategies for tracing metabolic signatures to elucidate diseases and subsequently reorienting macrophage fate for immunotherapy targeting macrophage-centered diseases.

**Fig. 1:**
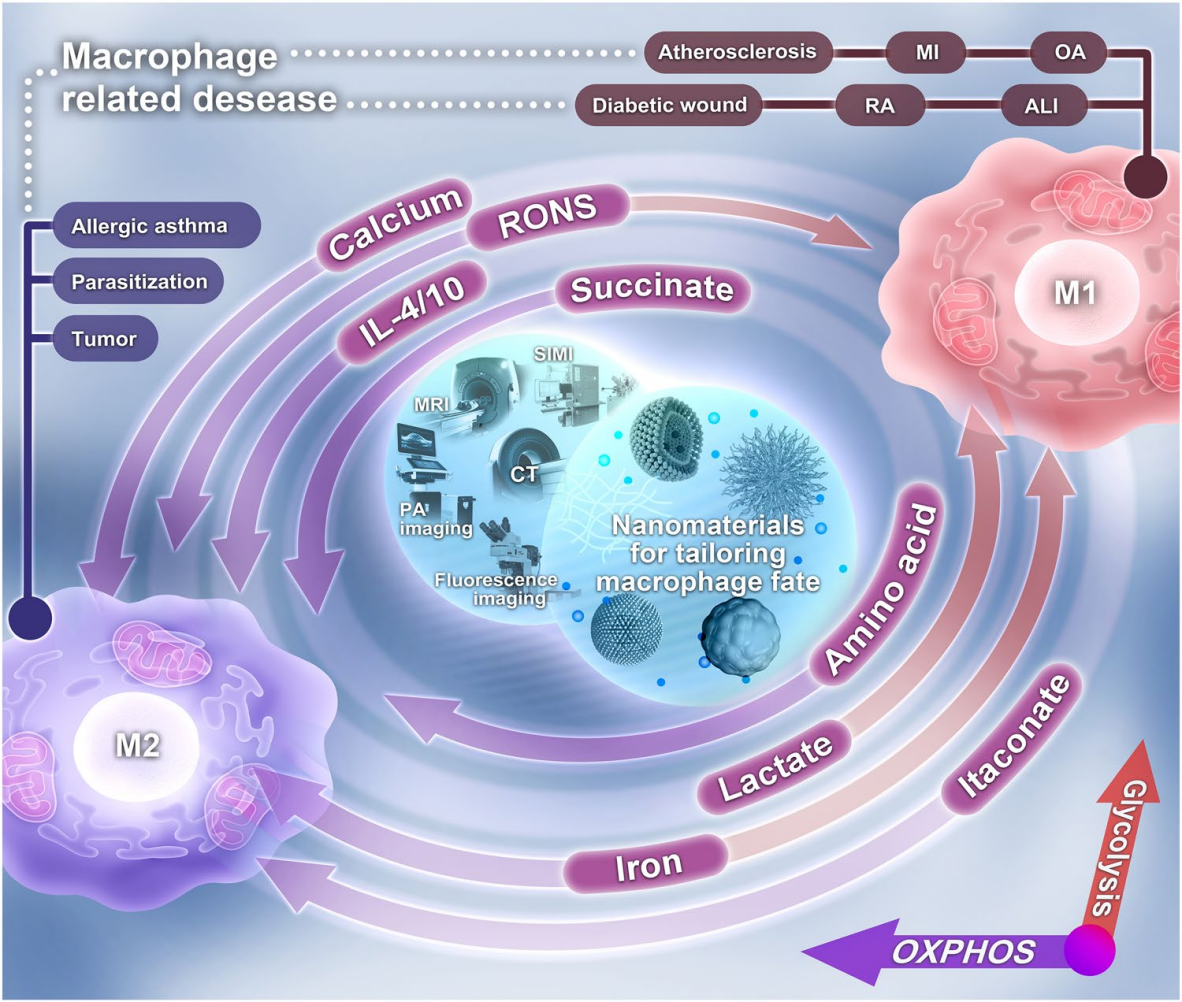
A systematic illustration of NM-based repurposing of macrophage metabolism and its applications

Here, we review recent advances in NM-mediated macrophage immunotherapy from a metabolic perspective (Fig. 1 and Table 2). We first provide a comprehensive overview of differentiated metabolism in polarized macrophages, followed by the relationship between metabolism and disease. Next, we systematically summarize the visualization of macrophage behavior in situ using NMs to reveal disease progression, as well as NM-mediated metabolic rewiring to re-educate macrophage fate for disease therapy. This review could serve as a versatile toolbox for researchers in various fields. Finally, we discuss the challenges and future research directions in exploiting NMs to reprogram macrophage metabolism. We anticipate that this review will contribute to advancing the understanding of research progress in regenerative medicine materials mounted on macrophage immune responses by modulating metabolism, guiding the reasonable design of immunomodulatory biomaterials to re-educate macrophage responses, and driving the application of macrophage immunotherapy in the field of immune engineering.

**Table 2:** NMs repurpose cell metabolism to reorient macrophage fate and their applications

Disease types	Polarized direction	Targets and strategies	Potentially polarized mechanism	Application	References
M1-related	M1-to-M2	RONS, Scavenging RONS	HA@RH-CeO_X_ scavenges ROS and inhibits TLR4 signaling pathway	RA	[159]
			MFC-MSNs produce O_2_ and scavenge ROS	RA	[155]
			HTON produces H_2_ to mediate ROS scavenging	RA	[163]
			PNSG scavenges NO and evokes H_2_S with ROS scavenging ability	RA	[171]
			NAHA-CaP/siRNA releases NO scavenger to eliminate NO and CA9siRNA to silence CA9 expression	OA	[172]
			CAT&SMT@ZIF-8 inhibits NOS activity and catalyzes O_2_ production	OA	[173]
			NOArg-PEA reduces NO by re-equilibrating NOS metabolic pathways	Diabetic wound	[174]
			DEX@PPNP scavenges excess ROS/NO and releases DEX to block inflammatory signals	OA	[175]
			DS/TPP-MMSP releases MnO_2_ to eliminate ROS and SMT to inhibit NOS for NO production	OA	[20]
		Succinate, Inhibiting SDH	Disodium malonate inhibits SDH activity to block mtROS production	Reperfusion injury	[176]
		Itaconate, Supplying itaconate or derivatives	ITA-OD/HD/DD releases ITA to exert anti-inflammatory abilities	Inflammatory diseases	[177]
			PCL/DMI also releases DMI to suppress IL-23/17 axis and promote Nrf-2	MI	[178]
		Iron, Depleting iron	P1-3 with DFO and catechol units chelate iron and scavenge iron-induced ROS	ICH	[179]
			P(MMD-co-LA)/DFO captures iron to inhibit ROS formation	Nerve regeneration	[180]
		IL-4, Supplying IL-4	MGC/IL-4 pDNA scavenges ROS and delivers IL-4 pDNA	MI	[181]
			PM-MG-CPIL4 releases IL-4 to initiate IL-4 signaling pathway	Ischemic stroke	[182]
		IL-10, Supplying IL-10	PF-IL-10 releases IL-10	Skin regeneration	[183]
			IL-10^+^ EVs release IL-10 to inhibit mTOR signaling	AKI	[184]
		Calcium, Depleting Ca^2+^	UCNP@mSiO_2_ releases Ca^2+^ regulators to supply or deplete [Ca^2+^]i	Immune diseases	[185]
			PPTB NPs release BAPTA-AM to glut excess [Ca^2+^]m to reduce mtROS production	Periodontitis	[75]
			METP NPs release EGTA to block Ca^2+^ influx and inhibit HIF-1α/ROS production	OA therapy	[186]
			BCP/MVS triggers [Ca^2+^]i outflow and inhibits ERK1/2 pathway	Bone regeneration	[187]
			FA − PEG − R − NPs@siERN1 deliver siERN1 to interfere with IP3R1/3 pathway to reduce [Ca^2+^]i	RA/IBD therapy	[188]
		Amino acid	Au@ZIF-8 enhances GS activity to elevate glutamine and alter other metabolic pathways	Metabolic diseases	[189]
			PEKK-L reemerges MET signaling to modulate Arg and energy metabolism	Bone regeneration	[190]
M1-related	M2-to-M1	RONS, Inducing RONS production	tTDCR NPs burst ROS	Cancer	[164]
			DMON-SNO mediates NO production	Cancer	[166]
		Iron, Delivering iron	SPF releases iron to induce oxidative stress	Cancer	[191]
			PEG-Fns release iron to induce ROS production	Cancer	[192]
			HION@Macs degrade into iron to activate the NF-κB pathway	Cancer	[193]
		Amino acid	DON blocks glutamine metabolism to upregulate M1 TAMs	Cancer	[194]
		Lactate, Metabolizing lactate	DL@NP-M-M2pep delivers DL to block PI3K/Akt and activate the NF-κB pathway	Cancer	[195]
			Lox-loaded Gd/CeO_2_@ZIF-8 depletes lactate	Cancer	[196]
			*E. hallii*-loaded E@Fe-DOX metabolizes lactate to butyrate	Cancer	[197]

## Macrophage Metabolism: An Underlying Target for Tailoring Macrophage-Centered Diseases

**Fig. 2:**
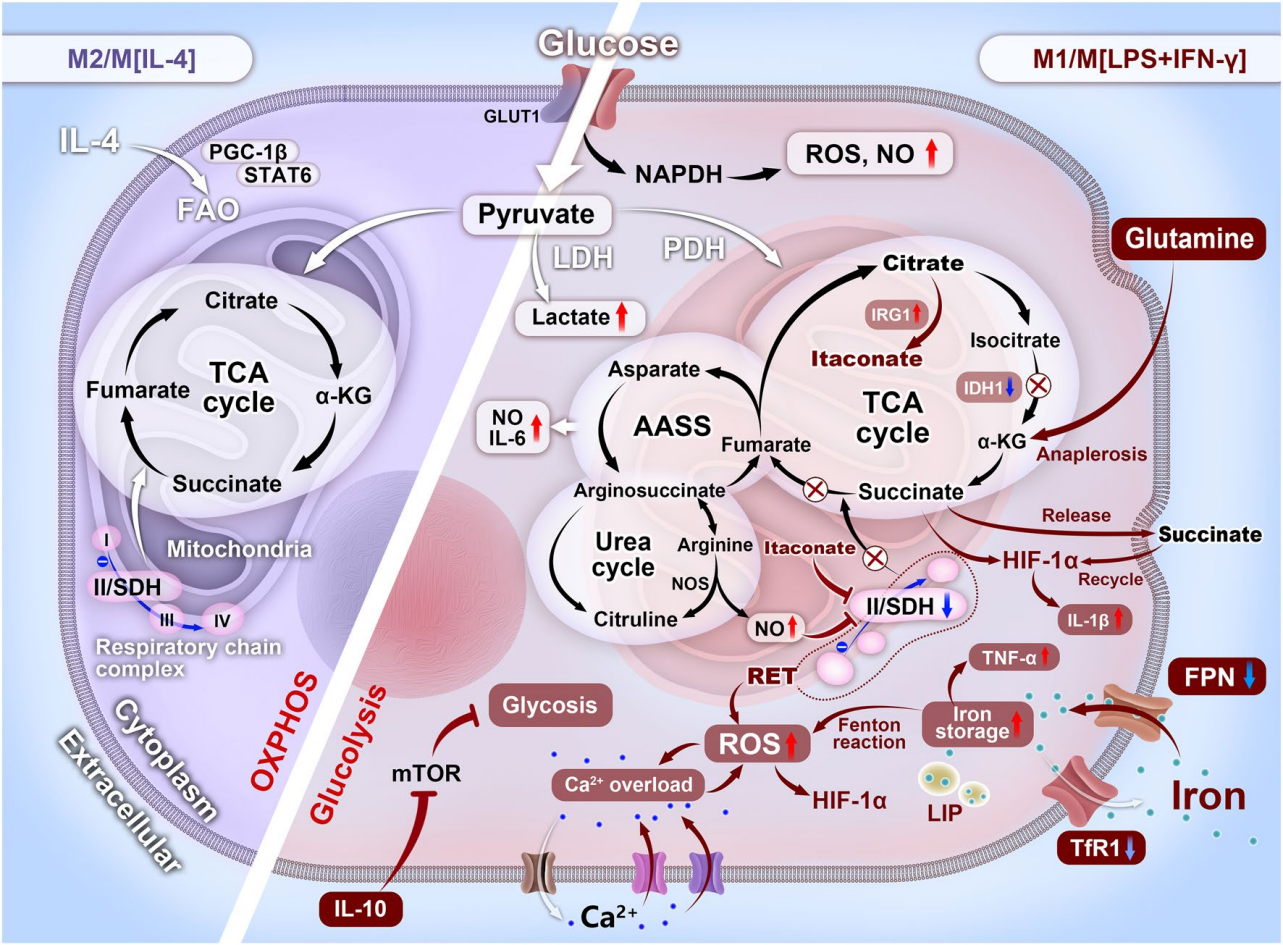
Metabolic adaptation in M1 and M2 macrophages in response to environmental cues: an underlying target for macrophage theranostics

The metabolic reprogramming of macrophages is strongly associated with their polarization [44–46] (Fig. 2). Macrophages coordinate their own response to oxygen gradient [8]. Contrary to M2, which have an intact TCA cycle that maintains a sustained supply of ATP (~ 30 per glucose) through OXPHOS and FAO to improve tissue repair, M1 macrophages substantially undergo enhanced glycolytic metabolism and impaired OXPHOS to adapt to hypoxia. Glycolysis produces less ATP (~ 2 per glucose) at a faster rate than OXPHOS and provides metabolic intermediates required for the biosynthesis of nucleotides, amino acids, and fatty acids [47].

LPS(+ IFN-γ)-activated M1 macrophages upregulate glucose transporter-1 (Glut1) to enhance glucose uptake [48] and upregulate glycolytically rate-limiting enzymes such as pyruvate kinase M2 (PKM2) to activate the glycolytic pathway. This pathway generates pyruvate to fuel the TCA cycle and produces lactate by lactate dehydrogenase (LDH)-catabolized pyruvate [49]. PKM2 enhances IL-6 and IL-1β secretion by phosphorylating signal transducer and activator of transcription (STAT) 3 and induces inflammasome-mediated pro-IL-1β and IL-1β secretion through HIF-1α activity [50, 51]. Moreover, NAPDH derived from upregulated PPP in M1 macrophage overproduced ROS via NADPH oxidase [24, 52].

Two metabolic breakpoints occur in the truncated TCA cycle of M1-polarized macrophages [14, 15, 53]. The first breakpoint occurs at the downregulated isocitrate dehydrogenase (IDH) 1, which converts isocitrate into α-ketoglutarate (α-KG) in M2, resulting in citrate accumulation, facilitating the biosynthesis of fatty acids and prostaglandins in M1 macrophages [12]. Glutamine enters the cytosol via the ASCT2/SN2 transporter and is metabolized to glutamate via glutaminase, followed by forming α-KG to fuel the TCA cycle occurred at interrupted α-KG production caused by IDH1 silence, and is termed glutamine anaplerosis [54]. The accumulated citrate is metabolized by citrate lyase into oxaloacetic acid and acetyl-CoA (precursors for ROS and NO, respectively) after transport to the cytosol via citric acid transporter [14, 15]. Intriguingly, immune-responsive gene 1 (IRG1) redirects citrate to itaconate, which synergizes with NO to block succinate dehydrogenase (SDH), forming the second breakpoint, which accumulates inflammatory succinate [14]. The succinate receptor, G protein-coupled receptor-9 (GPR91), recognizes extracellular succinate from M1 macrophages to form a feedforward loop to induce macrophage activation [55, 56].

The broken TCA cycle is co-opted to form an aspartate–argininosuccinate shunt (AASS) that feeds the TCA cycle at fumarate and replenishes the cycle after the breakpoint at SDH. AASS links the TCA cycle to the urea cycle, a metabolic pathway closely involved in NO production [13, 15, 16]. Intriguingly, blocking the rate-limiting enzyme of AASS, aspartate arginine succinate shunt enzyme, inhibits NO/IL-6 production and inducible nitric oxide synthase (iNOS) expression, both of which are biomarkers for M1 [5].

Amino acids feed the TCA cycle and compensate the level of intermediates in this pathway [13, 16]. Arginine is catabolized via the iNOS/Arg-1 axis. In M1 macrophages, upregulated iNOS metabolizes arginine to citrulline and NO [45, 57]. Recycled citrulline synergizes with aspartate to synthesize arginine for NOS reuse in turn by upregulating argininosuccinate synthase and argininosuccinate lyase in M1 macrophages through the citrulline–NO cycle [58]. The initial NO burst in M1 derives from extracellular arginine [59]. Multiple pathways-produced NO nitrosylates iron–sulfur-containing proteins to inhibit the electron transport chain (complexes I, II, and IV), thereby impairing OXPHOS action (e.g., electron transport, ATP production) against bacteria and tumor or causing other diseases [60, 61]. Repurposing mitochondria to produce mitochondrial ROS (mtROS) is an outcome of impaired OXPHOS [62]. Conversely, elevated Arg-1 in M2 competes with the iNOS-arginine pathway and hydrolyzes arginine to polyamines, supporting tissue remodeling [58, 63]. Polyamine production initiates M2 polarization [64].

Macrophages regulate iron metabolism. Physiological iron uptake primarily relies on heme oxygenase (HO)-mediated iron recycling, predominantly stores in labile iron pool (LIP) by binding ferritin (Ft), and releases iron through transferrin receptor 1 [65]. Ferroportin (FPN)-excreted free iron avoids oxidative damage caused by iron-induced Fenton reaction [66, 67]. Iron metabolism defines macrophage subsets, with M1 macrophages increasing iron retention to exhibit an iron-sequestering phenotype by upregulating ferritin and hepcidin and downregulating TfR1, FPN, and HO-1 expression, compared to M2 macrophages, which exhibit reduced iron levels (termed the iron-releasing phenotype) [68–70]. Iron deprivation in human macrophages directly blocks the transcription of respiratory chain enzymes, such as Fe–S clusters, and increases glucose-derived citrate pools related to lipid accumulation, impairing OXPHOS and increasing glycolytic flux to activate the M1 phenotype. Iron overload promotes ROS production and deviates glycolysis to activate the M1 response. Acute iron chelation in LPS-activated macrophages increases the itaconate/succinate ratio to decrease IL-1β and TNF-α secretion, thus blocking M1 polarization [71].

Calcium homeostasis determines macrophage phenotypes. Excess Ca^2+^ stimulates the M1 response. Cytosolic Ca^2+^ concentration ([Ca^2+^]c) is maintained 1000 times lower than the extracellular level through several Ca^2+^ outflow-mediated pathways in resting cells [72, 73]. Transient Ca^2+^ influx facilitates OXPHOS and ATP production, whereas prolonged influx disturbs several dehydrogenases of the TCA cycle and ATP synthase and triggers increased opening of mitochondrial permeability transition pores (mPTPs), which damages OXPHOS to burst ROS and form a positive feedback loop between Ca^2+^ and ROS, where ROS enhances Ca^2+^ influx and vice versa, initiating glycolysis to activate the M1 phenotype [74, 75].

Consistent with their opposing immune responses, M1-polarized macrophages undergo a large-scale metabolic rewriting, accompanied by various metabolite changes compared with M2 macrophages [76, 77], which can be summarized in the following categories: (1) Interrupted TCA cycle, upregulated PPP, and other alterations damage OXPHOS, triggering ROS production; (2) The second metabolic breakpoint induces succinate accumulation and then burst of ROS via SDH-mediated succinate oxidation; (3) Enhanced IRG1 redirects citrate to itaconate, thus inhibiting SDH-mediated succinate oxidation to decrease ROS production; (4) Compensatory AASS and urea cycle are responsible for NO production; (5) Increased iNOS competes with Arg-1, metabolizing arginine into NO; (6) Glutamine anaplerosis forms α-KG to fuel the first breakpoint at IDH1; (7) Persistent Ca^2+^ influx-induced [Ca^2+^]i overload impairs mitochondrial function to produce mtROS; (8) Iron overload triggered by unbalanced iron transport leads to oxidative stress via iron-induced Fenton reaction; (9) IL-4 links anti-inflammatory function with upregulated FAO to support the M2 response; (10) IL-10 blocks the mTORC1 pathway and lowers the iNOS/Arg ratio to inhibit glycolysis; and (11) Lactate depletion orients toward the M2 phenotype despite contradictory conclusions.

Leveraging these metabolic parameters as underlying targets could guide the design of biomaterials for treating macrophage-driven diseases.

## NM-Mediated Macrophage Activity Visualization and Fate Tailoring Based on Metabolic Parameters

**Fig. 3:**
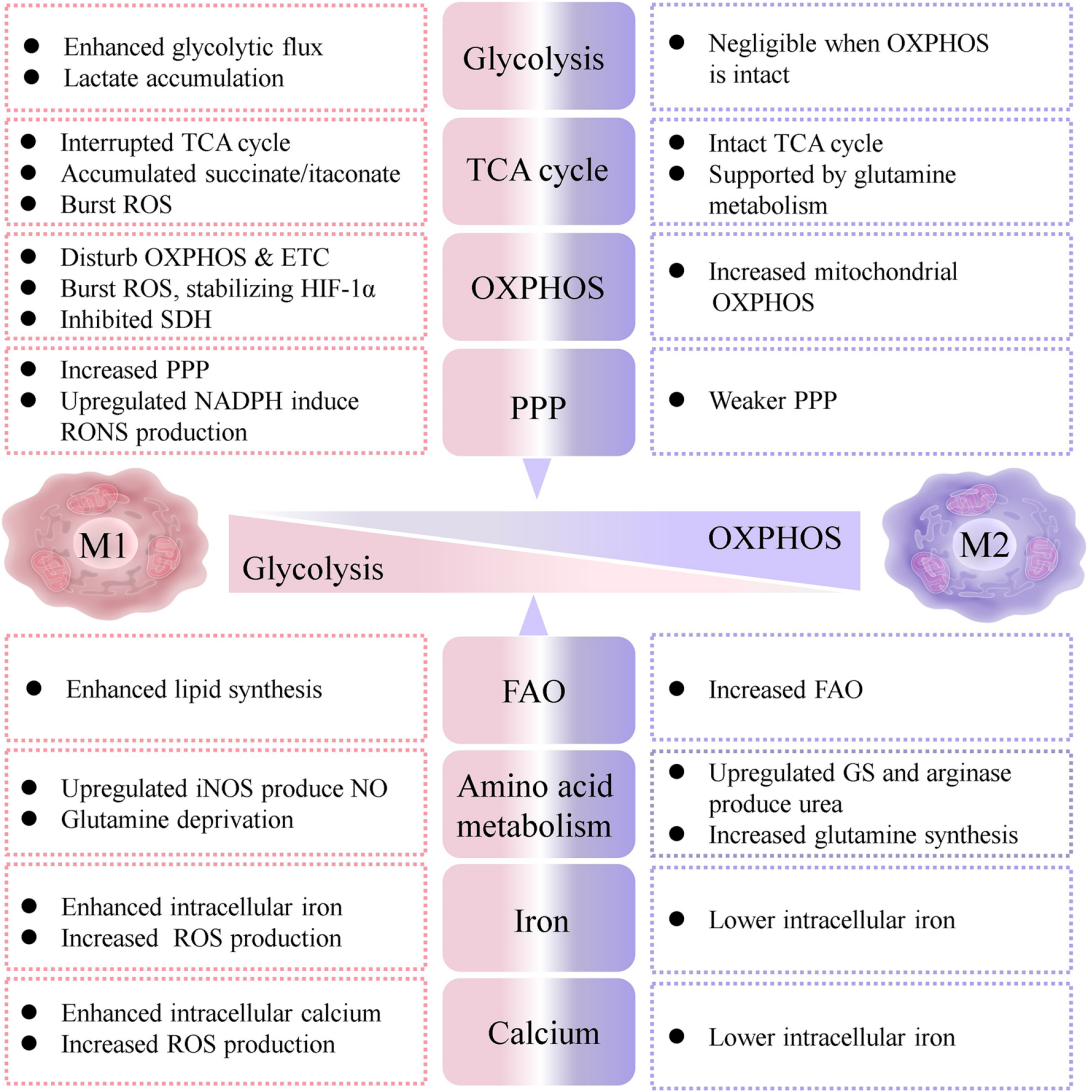
Schematic of distinguishable metabolic signatures of macrophage phenotypes

According to the distinguishable metabolism in M1/M2 macrophages (Fig. 3), the subsequent sections in this article will systematically elaborate NM-traced macrophage behaviors relying on multiple imaging techniques and reorient the macrophage phenotype to tailor its fate for immunotherapy.

### RONS

#### NMs Visualize RONS to Evaluate Macrophage Activity

ROS are considered a series of chemically active substances generated from intracellular oxygen through electron transfer, primarily including hydrogen peroxide (H_2_O_2_), hydroxyl radical (OH·), hypochlorous acid (HOCl), and superoxide anion (O_2_·^–^) [78]. Under stimuli, resting macrophages are polarized to the M1 phenotype with glycolysis, a mode of increased ROS production [11], whereas the M2 phenotype favors OXPHOS to maintain the physiological activity of cells with less ROS production [79]. ROS act as a double-edged sword in modulating macrophage polarization [80, 81]. At low levels, ROS are beneficial for initiating macrophages with M2, whereas at high levels, ROS drive macrophage M1 polarization [82, 83].

With the help of interdisciplinary strategies, a well-designed fluorescent “off–on” framework is opening the door for real-time imaging of living cells [84–87]. The traditional ROS imaging strategy involves applying fully- or semi-synthetic dyes, which emit fluorescence when oxidized by ROS [88]. H_2_O_2_ is the most stable and abundant endogenous ROS and acts as the precursor for producing other ROS at concentration ≥ 1 mΜ [89, 90], such as HClO [91]. Mao et al. [92] designed a ratiometric two-photon quinolone skeleton-based probe via covalent cross-linking, containing oxathiolane as the HClO identification part, which rapidly responded to HClO by oxidizing oxathiolane in macrophages, accompanied by a shift in absorption peak from 492 to 563 nm, thus monitoring overgeneration of HClO in macrophages. This probe has unparalleled advantages, such as less light drift and background signal and higher spatiotemporal resolution than single fluorescence-based probes. Semi-synthetic dyes seem to be more attractive due to their simple synthesis from commercially available dyes, which are used for ROS imaging in macrophages, such as IR-780 iodide [93], 1,8-naphthalimide [94], and fluorescein [95].

**Fig. 4:**
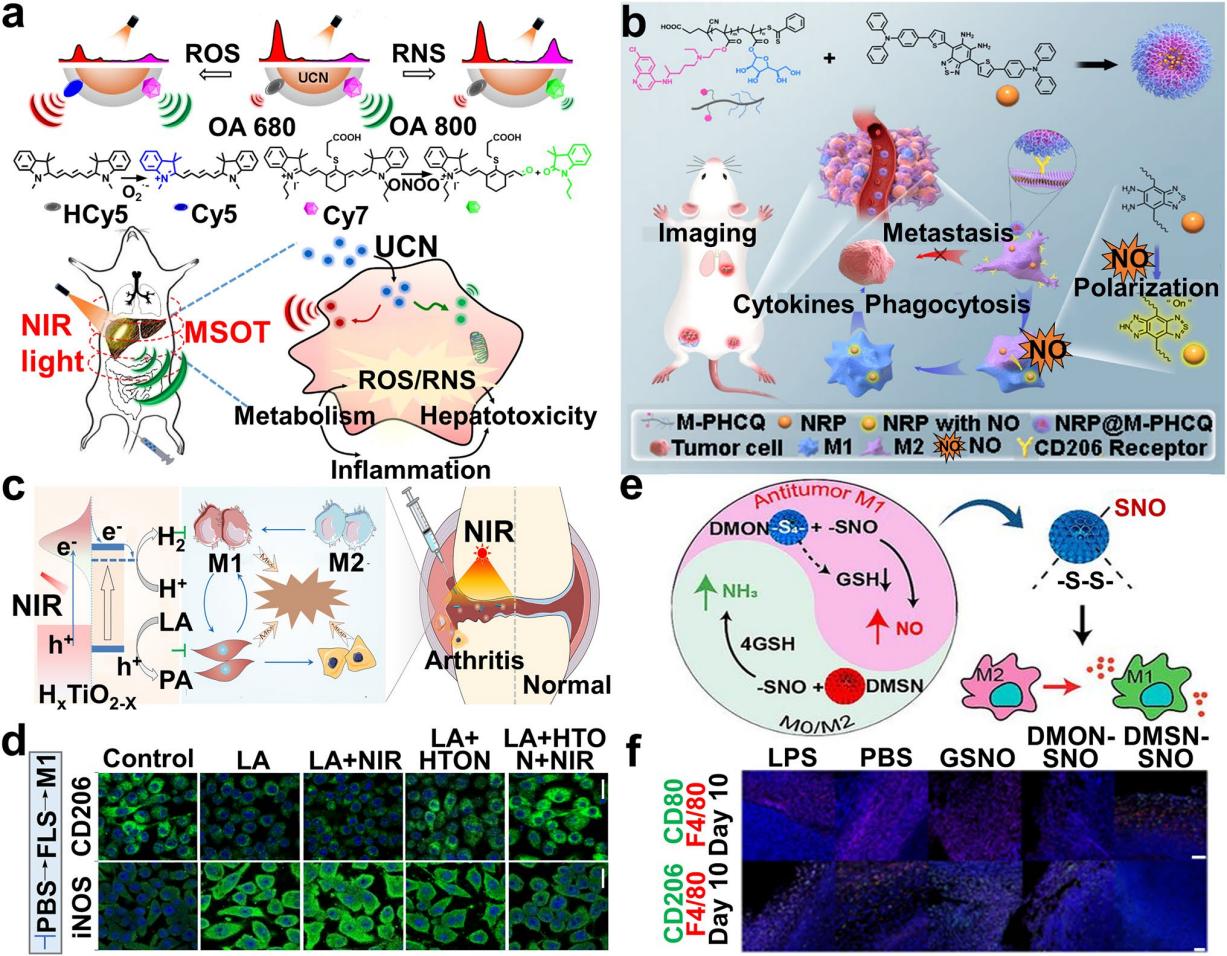
**a** NIR-responsive UCN visualizing endogenous RONS biomarkers in vivo [98]. Copyright 2019, Springer Nature. **b** Schematic of NRP@M-PHCQ fabrication, NIR-II imaging, and TAM repolarization based on secreted NO [121]. Copyright 2023, American Chemical Society. **c** NIR photocatalytic regulation of arthritic synovial microenvironment with HTON. **d** The effect of HTON photocatalysis on LA/FLS-stimulated macrophages [163]. Copyright 2022, American Association for the Advancement of Science. **e** DMON-SNO releases NO to modulate macrophage polarization. **f** IF staining of F4/80^+^ CD80^+^ and F4/80^+^ CD206^+^ cells in tumor tissues on day 10 after NPs injection [166]. Copyright 2022, American Chemical Society

Photoacoustic (PA) imaging with high spatial resolution and deep tissue penetration has been applied to track macrophage activity [96, 97]. Xing et al. [98] embedded ROS-responsive hydrocyanine substrate (HCy5) and RNS-responsive cyanine substrate (Cy7) onto lanthanide-doped nanocrystal surface via hydrophobic interactions to construct core–shell upconversion nanoprobes (size ~ 100 nm). These combined with multispectral photoacoustic tomography could ratiometrically trace RONS in RAW 264.7 cells due to the structural rearrangement or degradation of HCy5/Cy7, accompanied with altered absorbance at 640 or 800 nm, respectively, thus revealing the pathophysiological process in LPS- or acetaminophen (APAP)-induced inflammatory mice (Fig. 4a). The strategy monitors endogenous ROS/RNS distribution and alterations, mapping out the pathological process to advance high-throughput drug screening. Limited by the poor penetrability and stability of probes in vivo [86, 99], MRI has received more attention in cell imaging due to its high spatial resolution, almost zero background signal, and unrestricted signal penetration depth [100]. Our group [84] synthesized Mn-doped mesoporous silica nanoparticles (NPs) (diameter 110 nm) by an in situ hydrothermal method, which rapidly released Mn^2+^ once exposed to ROS to enhanced MRI signal. Thus, ROS imaging could be performed in macrophages derived from acute liver failure and acute pancreatitis mice. The strategy indicates that macrophage-centered diseases can be monitored using MRI-mediated ROS imaging. Sensor technology has broad prospects in macrophage imaging due to its high sensitivity, simplicity, speed, and cost-effectiveness [101]. Kumar et al. developed WaveFlex-structure sensors to monitor various biomarkers [102–107], such as alanine aminotransferase [108], providing valuable reference for tracking metabolic parameters through well-designed sensors.

Macrophages defend themselves against NO damage by forming a storage or transport system, but this protective mechanism may fail in an inflammatory state [14]. LPS(+ IFN-γ)-activated M1 macrophages are characterized by significantly elevated NO, which is 30-fold higher [20, 109] than that in M2 macrophages [8, 45, 61], where NO induces nitrative stress [110]. Recently, exploiting probes to timely image NO for tracking macrophage behavior has attracted great attention, such as the *o*-diaminobenzene unit [111–113], *N*-nitrosation of secondary amines [114–117], and reductive deamination [118, 119]. Nagano et al. did pioneering work in NO imaging probes by introducing *o*-diaminobenzene [111, 120]. The unit has flourished among several researchers. Yuan et al. [121] first synthesized an NO-responsive NIR-II probe with an *o*-diamine unit (NRP) by multiple cross-coupling reactions and loaded it into amphiphilic block copolymer to self-assemble nanoprobes (NRP@MPHCQ) with a diameter of 34.8 nm. The secreted NO in LPS + IFN-γ-induced RAW264.7 cells oxidized the NRP unit from a weak electron acceptor into a stronger acceptor along with increased absorbance at 600–1000 nm, enabling NRP@M-PHCQ to visualize the migration and polarization of M2 macrophages for tracking early 4T1 cell-bearing tumor metastasis (Fig. 4b). This approach specifically targets M2 macrophages and tracks their polarization with noninvasiveness and deep penetration and provides insights for detecting and inhibiting early tumor metastasis. To overcome the limitations of *o*-diamine units, *N*-nitrosifying secondary amines were introduced in the following research [122, 123]. NO further interacts with O_2_·^–^ to produce a more cytotoxic peroxynitrite (ONOO^–^), with an aberrant formation rate (6.7 × 10^9^ M^−1^ S^−1^) in the inflammatory microenvironment [78, 122, 124]. Researchers have developed a series of nanoprobes for imaging intracellular ONOO^–^ to unveil macrophages behaviors [125, 126].

To sum up, these imaging techniques have huge potential in noninvasively visualizing RONS in macrophages to reveal diseases progression. Cost-effective fluorescent technologies cannot avoid background interference, deviating from true results and hindering translation to the clinic. Clinic-utilized technologies, such as MRI, enable analysis of these metabolites with deep penetration and highly programmed accuracy to supply reliable pathologic information, but is limited by high-cost and time-consuming tests. The following questions are often posed in imaging analysis in vivo: (1) Do probes only accumulate in specific macrophages subtypes? (2) How to orthogonally image the dynamic changes of different RONS and tailor their relationship during pathophysiologic processes in real time? (3) Is the current single imaging modality for a single target susceptible to environmental interference? Multimodal probes simultaneously complete at least two imaging processes, which might break through the limitations of single imaging mode in vivo and integrate the advantages of various imaging techniques to achieve satisfactory imaging effects [127–129], perhaps serving as a banner to guide future research. Theoretically, intelligent probes must identify differences between macrophages and other cells, as well as differences in diverse macrophage subtypes, to visualize macrophage behaviors [130].

#### NMs-Educated Macrophage Fate Based on RONS

M1 macrophage activation is closely associated with upregulated TNF-α-mediated inflammatory pathways [131–133], such as the downstream mitogen-activated protein kinase (MAPK) and nuclear factor kappa-B (NF-κB) pathways [134]. Overall, RONS-scavenging downregulates inflammatory cytokine levels to modulate M2 polarization mainly through the following mechanisms: (1) Significantly reducing the nuclear translocation of p65 NF-κB by inhibiting IκB phosphorylation, which inactivates the NF-κB pathway to silence inflammatory genes, including iNOS [135, 136]; (2) Attenuating the phosphorylation of AMPK (ERK, JNK/SAPK, p 38), thus blocking the MAPK cascade; and (3) Inhibiting STAT-1 phosphorylation to deactivate the JAK/STAT-1 pathway [132, 137–139]. Conversely, RONS supplementation can stimulate these signaling pathway to deteriorate the M1 inflammatory response for tumor therapy [140].

Inspired by the dual role of RONS in macrophage activation, NM-mediated metabolic reprogramming, by regulating RONS levels to activate the required phenotype, has attracted increasing attention in disease therapy [141–146]. The methods that NM-manipulated RONS levels primarily focus on the following categories: (1) Natural antioxidases, such as glutathione peroxidase (GSH-Px) and superoxide dismutase (SOD) [147, 148]. Mao et al. [149] assembled SOD, catalase, and tannic acid (TA) into a spherical nanocomplex (TSC, ≈50 nm) via multiple noncovalent interaction between TA and proteins. TSC effectively maintained enzymatic activity and was positively targeted to mitochondria, which scavenged mtROS in H_2_O_2_-activated L02 cells and liver tissues (detected by MitoSOX or DHE probes) through the antioxidase cascade, thus restoring mitochondrial function and blocking the inflammatory cascade to exert hepatoprotective efficacy of APAP-induced liver injury and LPS/D-gal-induced acute hepatic failure mice, as measured by Annexin V-FITC/PI, flow cytometry, western blotting (WB), and H&E staining. This strategy develops a TA-based mitochondrial targeted nanoplatform rather than membrane potential-dependent, opening new paradigms in curing mitochondrial-related diseases. (2) Natural enzyme-mimicking synthetic NMs, such as Cu_5.4_O [150, 151], CeO_2_ [152–155], and Pt [156–158]. Hu et al. [159] mixed Ce^3+^ with the β-cyclodextrin-engineered hyaluronic acid (HA) framework to form ultrasmall CeO_x_ (3–5 nm) via the nucleation site of carboxylic groups, finally constructing spherical HA@RH-CeO_X_ micelles with a diameter of 200 nm, which accurately targeted LPS-induced RAW264.7 cells and released Ce with SOD-like enzymatic activity to scavenge ROS, detected by flow cytometry and confocal laser scanning microscopy (CLSM), thus downregulating inflammatory signals to drive M2 repolarization for incomplete Freund’s adjuvant (IFA)/collagen-induced RA mice therapy, measured by RT-qPCR, WB, CLSM, and immunofluorescence staining. The strategy resolves some challenges of CeO_x_-based nanozymes in regulating redox homeostasis, such as rapid clearance, and cytotoxicity caused by organic solvent-mediated nanozymes dispersion. (3) Antioxidative substances, including vitamin E/C, gases (e.g., H_2_, H_2_S), and polyphenols [147, 160, 161]. Hydrogen (H_2_) is considered an antioxidant because it selectively scavenges highly cytotoxic radicals (e.g., OH· and ONOO^−^) rather than the physiological ROS involved in cell signaling [162]. Zhao et al. [163] incorporated H_2_ into TiO_2_ nanorods to prepare monodisperse H_2_-doped TiO_2_ (H_x_TiO_2−x_) nanorods (HTON) by a full-solution method, achieving NIR-catalyzed H_2_ production due to a narrower bandgap of 1.45 eV, which scavenged ROS in LPS-induced RAW 264.7 cells and facilitated M2 repolarization, as detected by RT-qPCR, IF, and fluorescent staining, thus promoting photocatalytic synovial microenvironment regulation for complete Freund’s adjuvant (CFA)/collagen-induced arthritis (CIA) therapy in mice (Fig. 4c, d). The inexhaustible HTON enabled efficient NIR-photocatalysis on demand and continuously released H_2_ for long-term and reproducible therapy while avoiding toxic side effects. Conversely, excessive RONS reprogrammed TAMs from the M2 to M1 phenotype to ameliorate the immune tumor microenvironment (iTME) against tumor [140, 164, 165]. Yu et al. [166] modified tetrasulfide-bridged organosilica NPs with thiol and then generated *S*-nitrosothiol (SNO) by NaNO_2_-mediated thiol nitrosation, to synthesize porously dendritic nanostructured organosilica NO donors (DMON-SNO) with a size of ~ 230 nm, which increased NO delivery in IL-4-induced bone marrow-derived macrophages (BMDMs)/RAW264.7 cells, detected by Griess reagent and DAF-FM probe. The released NO could interfere with mitochondrial function in macrophages, thus activating M1 macrophages to hinder tumor growth in 4T1 cell-bearing mice. The macrophage phenotype was detected and quantified by flow cytometry, ELISA, WB, and RT-qPCR (Fig. 4e, f).

Each antioxidant method has its advantages and disadvantages, and its specific scenario should be considered when using it. Naturally occurring antioxidases are susceptible to the disease microenvironment, leading to inferior RONS-scavenging efficiency [167]. Antioxidative substances exhibit low ROS scavenging efficiency and poor bioavailability due to rapid metabolism and dissipative oxidation resistance [168]. Strategies for regulating RONS are mostly focused on synthetic metal-containing nanozymes with excellent antioxidative properties, which may potentially cause cytotoxicity due to undegradable metal elements and thus require further investigation. Green RONS-scavenging or supply platforms should be exploited to satisfy low toxicity, high biocompatibility, and sustainability issues. For example, the unique photosynthetic properties and easily functionalized surface of microalgae enable them to become an ideal candidate to construct a green RONS-scavenging platform [169] for disease therapy, such as diabetic wounds [170].

### Succinate Metabolism

In 2013, a comprehensive metabolic map profiling derived from Tannahill et al. revealed that sharp succinate accumulation in LPS-stimulated BMDMs directly damaged PHD activity to stabilize HIF-1α, which drives the expression of IL-1β and other HIF-1α-dependent genes, acting as an endogenous inflammatory signal to elucidate innate immunity [198, 199]. SDH, also termed complex II, is a hub that links OXPHOS to electron transport. Intriguingly, LPS-induced mitochondrial hyperpolarization and enhanced SDH-mediated succinate oxidation collectively repurpose mitochondrial metabolism, leading to ROS burst via reversed electron transfer (RET) by forcing electrons back into complex I. This process upregulates the proinflammatory gene profile to drive the M1 response [11, 200]. This phenomenon is reversed by potent SDH inhibitors, malonate derivatives, dimethyl malonate (DMM), which can rapidly hydrolyze to malonate intracellularly, blocking succinate-dependent ROS production and recovering pro- or anti-inflammatory gene homeostasis (IL-1β, IL-10, and IL-1RA) to remodel mitochondrial metabolism. This could be a valuable strategy to achieve M1 to M2 polarization. Valls-Lacalle et al. [176] revealed that pretreating reperfusion-injured mice with DMM in a concentration-dependent manner (0.03–30 mmol L^−1^) decreased the infarct size (24.57 ± 2.32% vs. 39.84 ± 2.78%) by blocking SDH-mediated ROS production and mPTPs. As an attractive target for regulating macrophage metabolism, unlike RONS, which have been extensively studied, researchers need to explore NM-modulated succinate oxidation to address this gap in our understanding of defense against diseases.

### Itaconate Metabolism

#### NMs Visualize Itaconate to Evaluate Macrophage Activity

Macrophages maintain a physiological pool of itaconate against inflammatory damage. The itaconate level in resting macrophages is as low as the micromolar level compared with > 1 mM in LPS-stimulated macrophages, possibly due to the elevated IRG1-mediated itaconate synthesis [201, 202]. In 2019, Wang et al. [203] introduced the Ac_4_ManNAz moiety to develop a thiol-reactive probe, 1-OH-Az, which systematically quantified and profiled itaconate-targeted cysteine sites in proteomes of RAW 264.7 cell lysates, in combination with quantitative chemoproteomic technology. They identified 260 itaconate-modified cysteines and found that itaconate inhibits glycolysis primarily by silencing glycolytic enzymes (e.g., ALDOA) to alleviate the inflammatory response in activated macrophages and revealed the negative feedback between glycolysis and the anti-inflammatory mechanism originating from itaconate. Nonetheless, this profiling method only indirectly monitors itaconate-modified cysteines in cell lysates [203]. Itaconate-alkyne bio-orthogonal probe (ITalk) was developed to directly enter cells [204] and achieve quantitative and site-specific chemoproteomic profiling of itaconate. They identified 1926 targets of itaconate involved in inflammation-related pathways and provided abundant data for understanding the role of itaconate in macrophages. However, the study was limited by itaconate-monitoring techniques (GC–MS) that failed to monitor the concentration distribution of itaconate in different intracellular regions. An innovative study designed a genetically encoded fluorescent biosensor (BioITA) to timely detect the itaconate dynamics curve at subcellular resolution in macrophages isolated from LPS-bearing mice [205]. The altered fluorescence intensity revealed itaconate fluctuations under inflammatory conditions, making it a powerful tool for imaging itaconate with temporal–spatial resolution in living macrophages.

Given that its anti-inflammatory efficiency, it is of great significance to develop biological probes to achieve itaconate-monitoring in macrophages to deepen our understanding of the unknown biological functions about itaconate. The current monitoring methods inevitably are dependent on liquid chromatograph mass spectrometer (LC–MS) and rarely directly obtain itaconate dynamic in macrophages, and more in-depth studies are needed to fill this relatively blank field.

#### NM-Educated Macrophage Fate Based on Itaconate

Itaconate (IA) is highly generated in stimuli-induced macrophages and mediates the cross-talk between immunity and metabolism as an anti-inflammatory metabolite to negatively regulate the inflammatory response and govern M2 polarization in vitro and in vivo via multiple mechanisms [206–208]: (1) It inhibits SDH activity by blocking RET-dependent ROS production at complex I, thereby inhibiting HIF-1α activity and IL-1β production, and subsequent, inactivating the NLRP3 inflammasome [11, 207]. (2) It alkylates residues of KEAP1 via Michael addition to upregulate the expression of Nrf-2, due to the presence of electrophilic α, β-unsaturated carboxylic acid [209]. (3) It induces electrophilic stress to inhibit IκBζ translation that regulates secondary transcription via upregulated ATF3 but not Nrf-2, described as the IκBζ–ATF3 inflammatory axis [210]. (4) It dephosphorylates IL-23, failing to bind to the IL-17 gene promoter to inhibit downstream IL-17 transcription [178]. (5) It inhibits the NF-κB pathway [211].

**Fig. 5:**
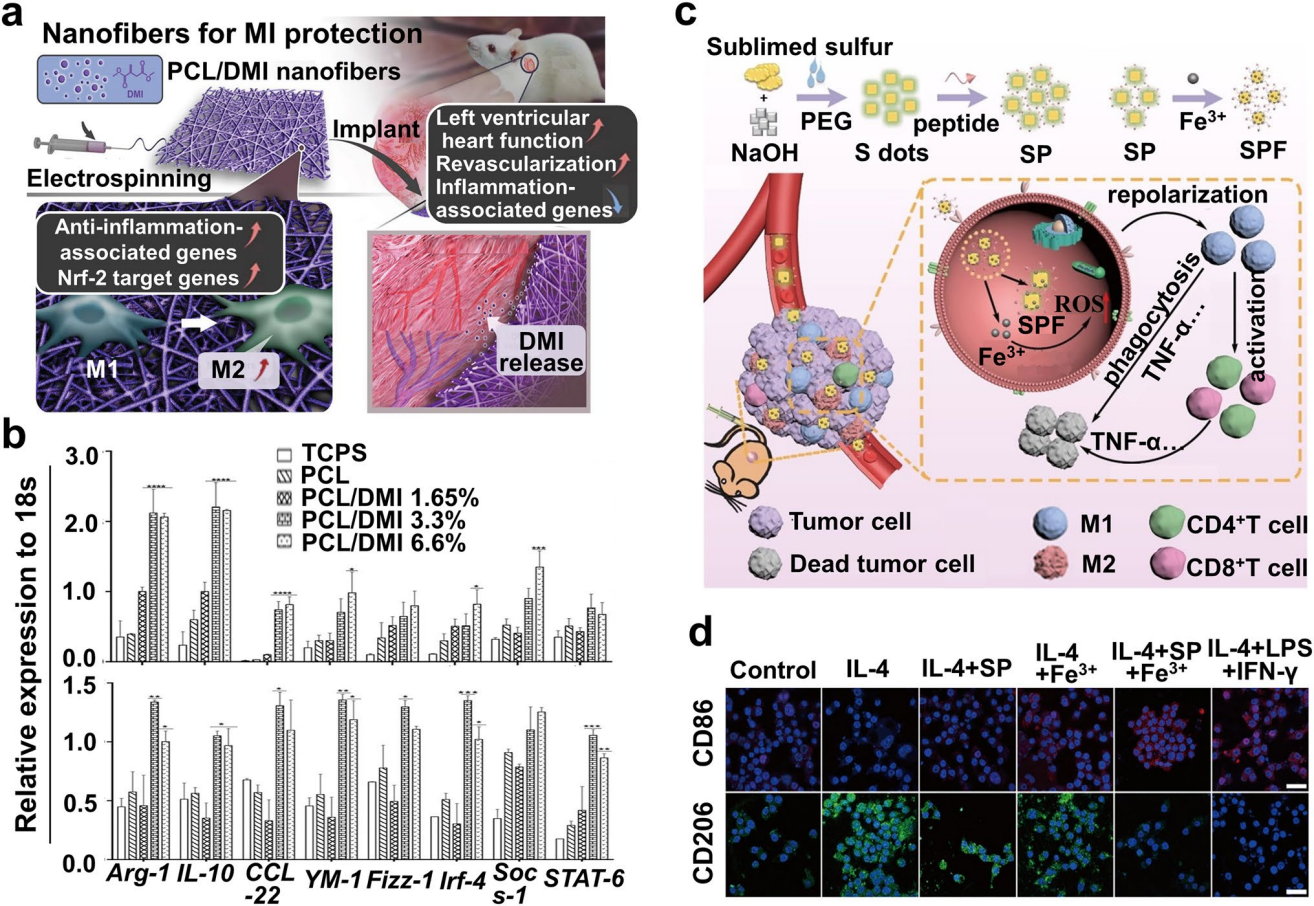
**a** Schematic illustration of DMI-loaded PCL nanofibers and modulating M1-to-M2 macrophage polarization. **b** The M2-related gene expression of RAW 264.7 cells (upper) and BMDMs (lower) after treatment with different groups for 24 h [178]. Copyright 2021, Wiley–VCH GmbH. **c** Schematic of SP/SPF formation and SPF reeducates TAMs from M2 to M1 for cancer immunotherapy. **d** IF staining of CD86 to M1 and CD206 to M2 in LPS-induced RAW 264.7 cells after different treatments [191]. Copyright 2021, American Chemical Society

The properties of itaconate provide a novel direction for exploiting immunometabolism-based disease intervention strategies. Mechanically, the strategy that rewrites itaconate metabolism for achieving additional benefits is primarily dependent on delivering itaconate into macrophages in a locally on-demand manner. Yu et al. [212] fabricated monodisperse hydrogel microspheres by the self-assembly of zinc ions, 2-methylimidazole, and IA (IA-ZIF-8@HMs) (diameter 20.25 ± 0.43 µm) via one-step microfluidic technology under ultraviolet light. These sustainably released itaconate under the acidic environment of lysosomes and exerted excellent anti-inflammatory and antioxidative stress damage in vitro and in vivo for mono-iodoacetic acid-induced OA mice therapy (analyzed by ELISA, H&E staining, and O-fast green staining). Nevertheless, limited by the poor cell permeability of itaconate, it is necessary to develop functionally similar itaconate derivatives (dimethyl itaconate [DMI] [177] and 4-octyl itaconate [4OI] [213]) or itaconate-loaded NMs. Nakkala et al. [178] synthesized DMI-encapsulated poly-ε-caprolactone nanofibers by electrospinning technology to downregulate the IL-23/IL-17 inflammatory axis-related genes via the IκBξ–ATF3 axis and upregulate the antioxidant Nrf-2 target genes in LPS/IFN-γ-stimulated RAW 264.7 cells and BMDMs, thereby rewriting itaconate metabolism to polarize the M1-to-M2 phenotype and relieve inflammation against MI (Fig. 5a, b). The percentage of M1/M2 macrophage in vitro or in vivo were assessed by ELISA, flow cytometry, IF, and PCR. The study unveils the potential metabolic regulatory function of DMI on macrophages in the tissue microenvironment.

However, these derivatives may be insufficient to mimic endogenous itaconate. No conclusive evidence confirms that their therapeutic action is derived from itaconate, and no real consensus exists about how to effectively deliver itaconate intracellularly [206]. The burgeoning field of delivering itaconate via biomimetic carriers to alter intracellular itaconate metabolism may be confronted with great development potential and challenges. Moreover, the role of itaconate in M1 macrophages has been unambiguously investigated, whereas the role of itaconate as an M2 macrophage inhibitor has been scarcely studied [214–216].

### Iron Metabolism

#### NMs Visualize Iron to Evaluate Macrophage Activity

Iron is an essential trace element that participates in multiple metabolic processes [217, 218]. Macrophages play a core regulatory role in iron homeostasis according to their unique genetic program [219]. Opposite iron metabolism operates in macrophages: iron is sequestered in M1 macrophages against bacterial infection [220], whereas it is released in M2 phenotype to facilitate tissue repair but may promote tumor growth [70, 221]. Tracking iron metabolism to evaluate macrophage activity to understand disease progression is a promising prospect and remains challenging.

Benefiting from noninvasiveness, spatiotemporal optical readout, and low-cost, fluorescent technologies have emerged to track iron homeostasis, such as LIP, which triggers Fenton reaction-induced oxidative stress and oppositely expresses in M1/M2 macrophages [222]. Xing et al. [223] designed a fluorescent probe by covalently coupling coumarin 343 (fluorophore) and 3-nitrophenylazanyl ester to visualize the activation of M1 and M2a macrophages by monitoring LIP levels via a photoinduced photon transfer mechanism induced by Fe(II)-activated N–O bond cleavage. This technology provides insights for imaging iron using fluorescent technologies under pathophysiological conditions. Macrophages containing abundant hemosiderin exhibit high MRI contrast. Limited by susceptibility to interference from background signals of fluorescence, MRI was utilized to investigate iron behaviors in macrophages [224, 225]. However, the slow imaging speed and high-cost limit its application. Secondary ion mass spectrometry (SIMS) was used to for iron analysis, even at low concentrations [226–228]. Lovric et al. developed SIMS imaging to explore the uptake and subcellular localization of iron in alveolar macrophages, providing discernment and therapeutic strategies on iron metabolism at the subcellular level to reduce the cytotoxicity of iron overload [228]. Although SIMS can track iron with high sensitivity and spatial resolution (up to 37 nm), it is a new technology and rarely used due to the need for expensive instrumentations and time-consuming testing, similar to MIR. Moreover, macrophage behaviors are dynamic, whereas current monitoring methods can only be fixed at a certain time and environment. How to dynamically monitor metabolites has not been fully investigated, which motivates researchers to develop methods for dynamically tracking them in biological systems.

#### NM-Educated Macrophage Fate Based on *Iron*

Iron overload activates M1 macrophages to fine-tune the innate immune response and iron deprivation may block M1 [71]. Generally, iron levels manipulate REDOX homeostasis intracellularly. Capturing excessive iron can inhibit ROS formation to drive M2 polarization by silencing inflammatory pathways (e.g., NF-κB) [229], whereas iron replenishment induces oxidative stress to activate pathways, such as NF-κB, upregulating the inflammatory cascade to initiate the M1 response [221, 230, 231].

Researchers have extensively investigated on shaping the macrophage phenotype by reprogramming iron metabolism, including iron-scavenging and supply, for curing macrophage-driven diseases [232]. Zhu et al. [179] designed dual-functional macromolecular poly(catechol) nanoscavengers loaded with deferoxamine via the self-assembly with a particle size of 36.2 ± 7.6 nm, simultaneously chelating excess iron and relieving iron-induced oxidative stress in RAW 264.7 cells, thus protecting mice with intracerebral hemorrhage (ICH). Iron levels were adopted via CLSM and flow cytometry (Perl’s staining). This delivery carrier addresses the dose-limiting toxicity using chelators alone and achieves long-term stable release, providing a clue for iron-depleting therapeutic regimen. Similarly, iron dextran therapy resolved kidney macrophage iron depletion by suppling LIP and downregulating TfR1, which polarizes macrophages toward M1 for chronic kidney disease therapy [233]. Much of the observed therapeutic effects of regulating iron metabolism can be traced to tumor therapy. Contrary to iron-scavenging driving the M2 phenotype against inflammation, repolarizing TAMs from M2 to M1 by replenishing iron is believed to be an attractive strategy against tumors [234, 235]. Sang et al. [191] modified S dots (synthesized by an O_2_-assisted top-down method) with TAM-targeted peptides to construct ultrasmall nanotraps (SP) with a size of 3.2 nm, which positively adsorbed and internalized endogenous iron into TAMs, attributed to the plentiful iron-binding sites on SP surface by coordinating with oxygen. SP-derived iron produced ·OH and NO in IL-4-induced BMDMs/RAW264.7 cells, detected by CLSM (·OH, DCFH-DA probe) and Griess kit (NO), which remodeled M2-to-M1 phenotype and formed a positive feedback loop between ROS production and M1 polarization, thus delaying 4T1-bearing breast tumor growth (Fig. 5c, d). The M2-to-M1-polarized behavior was detected through protein and gene levels by IF, flow cytometry, ELISA, WB, and immunohistochemical staining (HC). The nanotrap effectively targeted into TAMs and avoided excessive exogenous iron-caused cytotoxicity, providing opportunities to utilize endogenous substances against various diseases. Similarly, iron oxide NPs can be degraded to iron ions in the lysosomes of macrophages and induce oxidative stress to trigger the M1 response [193, 236, 237].

Iron regulator-loaded nanocarriers release regulators to manipulate oxidative stress intracellularly for disease therapy. However, uncontrollable release in other macrophage phenotypes or cells causes dose-dependent toxicity, which attributes to nonspecifically chelate physiologically essential iron. Modifying these nanocarriers with stimuli-responsive moieties may be a charming avenue to achieve targetable cellular internalization, drawn-out circulation half-time, long-term regional enrichment, and sustainable release [238]. It may be more in line with the on-demand release requirements in specific sites compared with direct physical release [192].

There are contradictory reports concerning its precise effects. Dietary iron overload in mice induces hepatic and peritoneal macrophage M2 polarization and inhibits the M1 phenotype [239]. Iron-incubated macrophages induce the translocation of NF-κB p65 nuclear and inflammatory cytokine expression, which is consistent with other studies [240, 241]. These contradictory findings illustrate the complexity of the impact of iron metabolism on macrophage polarization.

### Calcium Metabolism

Calcium is a crucial messenger that controls several cellular functions [242]. Prolonged Ca^2+^ influx impairs OXPHOS and phosphorylates certain enzymes (e.g., NOS), thereby facilitating the M1 response by stimulating NF-κB and MAPK pathways. Conversely, lowering intracellular calcium ([Ca^2+^]i) levels reduces mtROS production and inhibits ERK phosphorylation (MAPK-related), consequently downregulating proinflammatory factors and promoting M2 polarization [243–245]. Macrophage phenotypes link to [Ca^2+^]i levels, with elevated [Ca^2+^]i levels responding to M1 stimuli and reduced [Ca^2+^]i levels responding to M2 stimuli. Increasing attention has been focused on developing NM-regulated [Ca^2+^]i levels to remodel M1-to-M2 phenotype development for macrophage-driven immunotherapy [243, 246–249].

One strategy is to encapsulate Ca^2+^ regulators, such as EGTA [186] and BAPTA-AM [75], glutting excess [Ca^2+^]i, restoring ROS-mediated disordered mitochondrial homeostasis to inhibit M1 activation against Ca^2+^ overload-driven diseases. Kang et al. [185] loaded Ca^2+^ regulators (supplier DMNP-EDTA-Ca^2+^ or chelator BAPTA-AM) into mesoporous silica-decorated upconversion NPs to prepare UCNP@mSiO_2_ with a diameter of 58 ± 6 nm. This was further modified with the Arg-Gly-Asp (RGD) peptide-containing cap and photocleavable linker via cyclodextrin-adamantine host–guest complexation, which temporally enhanced or depleted [Ca^2+^]i (measured by Fura-2 AM probe) under NIR light by on-demand releasing Ca^2+^ regulators, remotely modulating RAW 264.7 cells with the M1 or M2 response to exert immune functions, as detected by IF, qRT-PCR. This system achieves intracellularly targetable uptake and time-dependent release. However, the underlying mechanism by which UCNP@mSiO_2_-resurged the M2 is unclear.

**Fig. 6:**
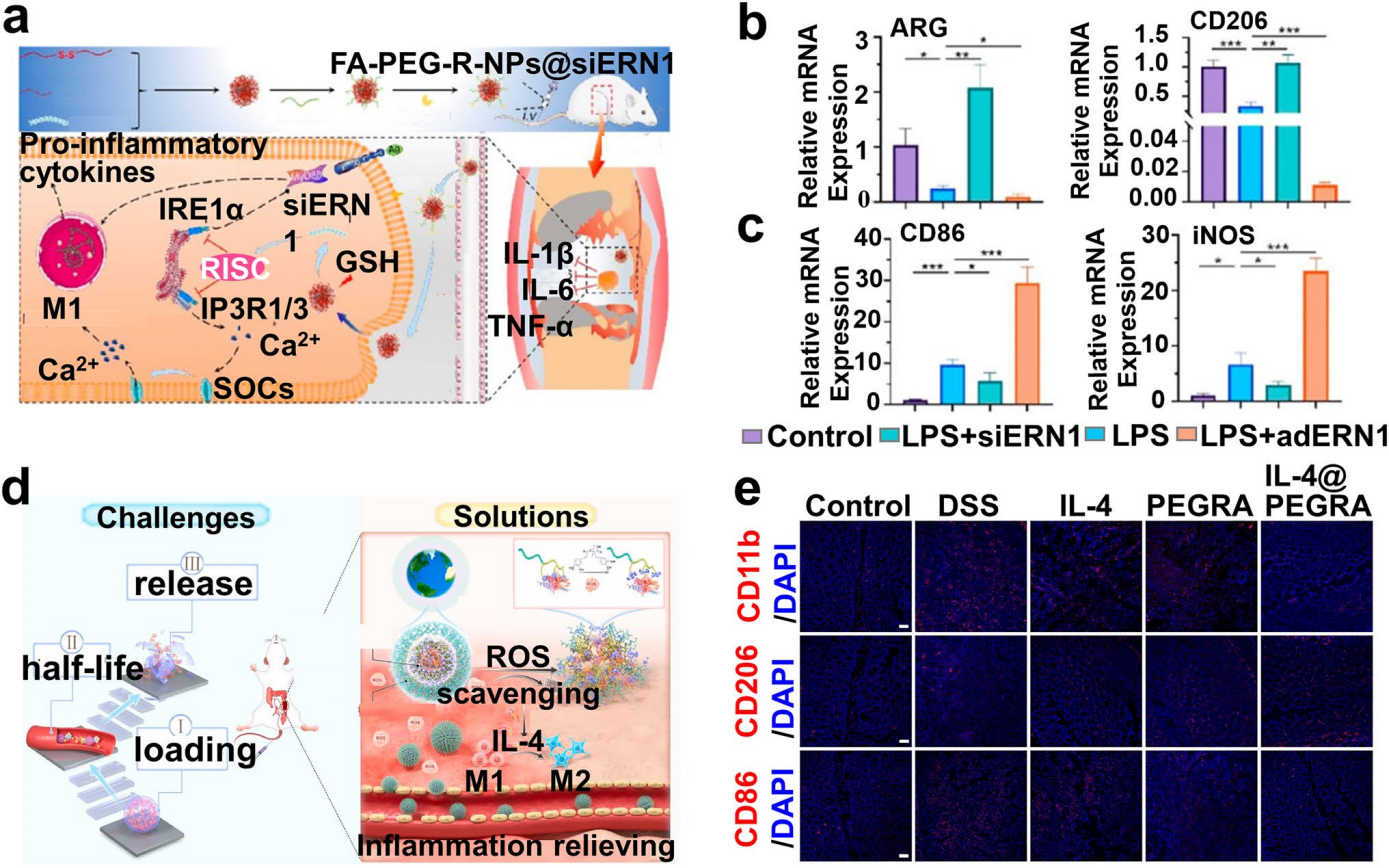
**a** Schematic diagram of delivered siERN1 promoting macrophage M2 polarization by interfering with IP3R and modulating [Ca^2+^]i. **b** M2 and **c** M1 marker expression levels detected via RT-qPCR [188]. Copyright 2021, American Chemical Society. **d** Schematic diagram of IL-4@PEGRA nanoarmor modulating M1-to-M2 polarization for IBD therapy. **e** IF staining of CD86^+^, CD206^+^, and CD11b^+^ cells in colon tissue derived from IBD-bearing mice on day 10 after treatment [262]. Copyright 2023, Elsevier

The other strategy is to block improper Ca^2+^ flow by interfering with related channels or signaling pathways [187]. Feng et al. [188] constructed a macrophage-targetable delivery system using polyethylenimine (PEI) and poly (β-amino amine) (PBAA) as siERN1 carriers (FA − PEG − R (Peptide: RKKRRQRRR) − NPs (ss-PBAA-PEI)@siERN1) with a diameter of ~ 110.0 nm through self-assembly, which released endoplasmic reticulum nucleus signaling 1 gene (siERN1) under pH 5.5/GSH to modulate [Ca^2+^]i levels (detected by flow cytometry and CLSM with fluo-4AM labeling) in LPS-stimulated RAW 264.7 cells by disturbing inositol 1,4,5-trisphosphate receptor 1/3 (IP3R1/3), thereby initiating M1-to-M2 polarization as demonstrated by WB, RT-qPCR to maintain immune homeostasis, demonstrating superior therapeutic efficacy in CIA and dextran sulfate sodium salt-induced IBD (Fig. 6a–c). This study confirms ERN1 as an effective target, providing promising insights into Ca^2+^-regulated siERN1 drugs design and its potential mechanisms.

Cross-talk between macrophage polarization and Ca^2+^ occurs in multiple diseases. Manipulating Ca^2+^ signaling can be regarded as an underlying adjuvant therapy. Akin to iron, Ca^2+^ regulator-loaded nanovehicles modulate intracellular oxidative stress by altering [Ca^2+^]i to fine-tune macrophage fate. The uncontrolled release within non-specific cells inevitably leads to dose-determined toxicity, as it does interference with ion channels or signaling pathways. Moreover, the accurate mechanisms underlying its functionality remain poorly understood. Strengthening mechanistic understanding of Ca^2+^ signaling provides more opportunities to develop Ca^2+^-based therapeutic approaches. Establishing an intelligent delivery system in terms of specific macrophage subpopulations by modifying targeted or stimuli-responsive moieties, achieving its oriented phagocytic uptake, prolonged regional enrichment, and spatiotemporal release as well as minimizing toxicity to adjacent cells remains a challenge.

### Interleukin-4/10

#### IL-4

The STAT pathway is associated with macrophage polarization [250, 251]. The IL-4-dependent STAT6 pathway responds to the M2 phenotype [9, 252–254]. In detail, IL-4 binds with its receptor to activate JAK2 downstream, inducing STAT6 phosphorylation and entering the nucleus to bind to promoter elements of IL-4-responsive gene, thus transcribing M2 lineage genes (e.g., *Il10*, *Arg-1*) to drive M2 differentiation, which acts as a vital immunoregulator to block inflammatory process [255–257]. Recently, IL-4 links anti-inflammatory potential to mitochondrial oxidative metabolism which was confirmed by Chawla et al. [77], who showed that IL-4-treated macrophages (known as M(IL-4)) strongly activated PGC-1β expression and upregulated FAO gene profiling (e.g., LPL) to promote M2 polarization, relieving macrophage-driven inflammation. Moreover, IL-4 and CSF-1 cooperatively enhance fatty acid synthesis (FAS)/FAO via the mTORC-IRF4 axis to support M2 polarization [258, 259]. Thus, M(IL-4) is closely associated with upregulated FAO.

Recognition of the anti-inflammatory potential of IL-4 paves the way for IL-4 supply from reasonable delivery platforms to drive macrophage polarization in some tissue engineering applications. Due to its short half-life of only several minutes and dose-dependent toxicity [260], multiple loading platforms through physical adsorption or encapsulation and chemical coupling have been developed to deliver exogenous IL-4 to cells or tissue for clinical application [182, 261]. Our group [262] developed a multilayered defensive platform (IL-4@PEGRA NAs) with a size of 96.45 ± 7.3 nm via the self-assembly of polycatechol-containing poly(ethylene glycol)-ylated phenolic rosmarinic acid (PEGRA) and IL-4, to preserve bioactivity and prolong circulation time. The platform delivered IL-4 into inflammatory tissues, achieving M1-to-M2 polarization in LPS-stimulated BMDMs and mice colon tissue in dextran sulfate sodium salt-induced acute colitis, thus protecting against IBD. The percentage of M1/M2 was measured by IF, flow cytometry, WB, and ELISA (Fig. 6d, e). The nanoarmor defended IL-4 against rapid biodegradation, prolonging circulation half-life, achieving triggered release, and avoiding immunogenic side effects. In addition to the aforementioned physical methods, Spieler et al. [263] genetically engineered an amino acid-incorporated IL-4 variant via genetic code expansion and then directly performed bio-orthogonal PEGylation (PEG-folate, a targeting moiety) by strain-promoted alkyne-azide cycloaddition to overcome the pharmacokinetic challenges of IL-4, which prolonged the half-life to 4 h in healthy mice and polarized primary murine macrophages toward M2 via RT-qPCR detection and accumulated into arthritic joints to cure antigen-induced arthritis mice.

#### IL-10

IL-10 initiates an anti-inflammatory response mainly dependent on the JAK–STAT3 pathway [264–266]. Briefly, IL-10 binds with IL-10 receptor to activate JAK1, which stimulates STAT3 to translocate into the nucleus, thus combining with specific DNA sequence to initiate DNA transcription and upregulate M2-associated genes (TGF-β1, Mrc1) for anti-inflammatory cascades. Recently, IL-10 was verified to remodel glycolysis and potentiate OXPHOS by blocking glucose uptake and glycolytic gene expression. IL-10 reprogrammed metabolic processes by inhibiting mTOR complex 1 (mTORC1) signaling via STAT3 signaling and accelerated mitophagy to eliminate impaired mitochondria, thereby controlling inflammation [17, 26, 267, 268]. IL-10 blocked NO production in activated macrophages by silencing iNOS [269, 270] and upregulating arginase [271].

**Fig. 7:**
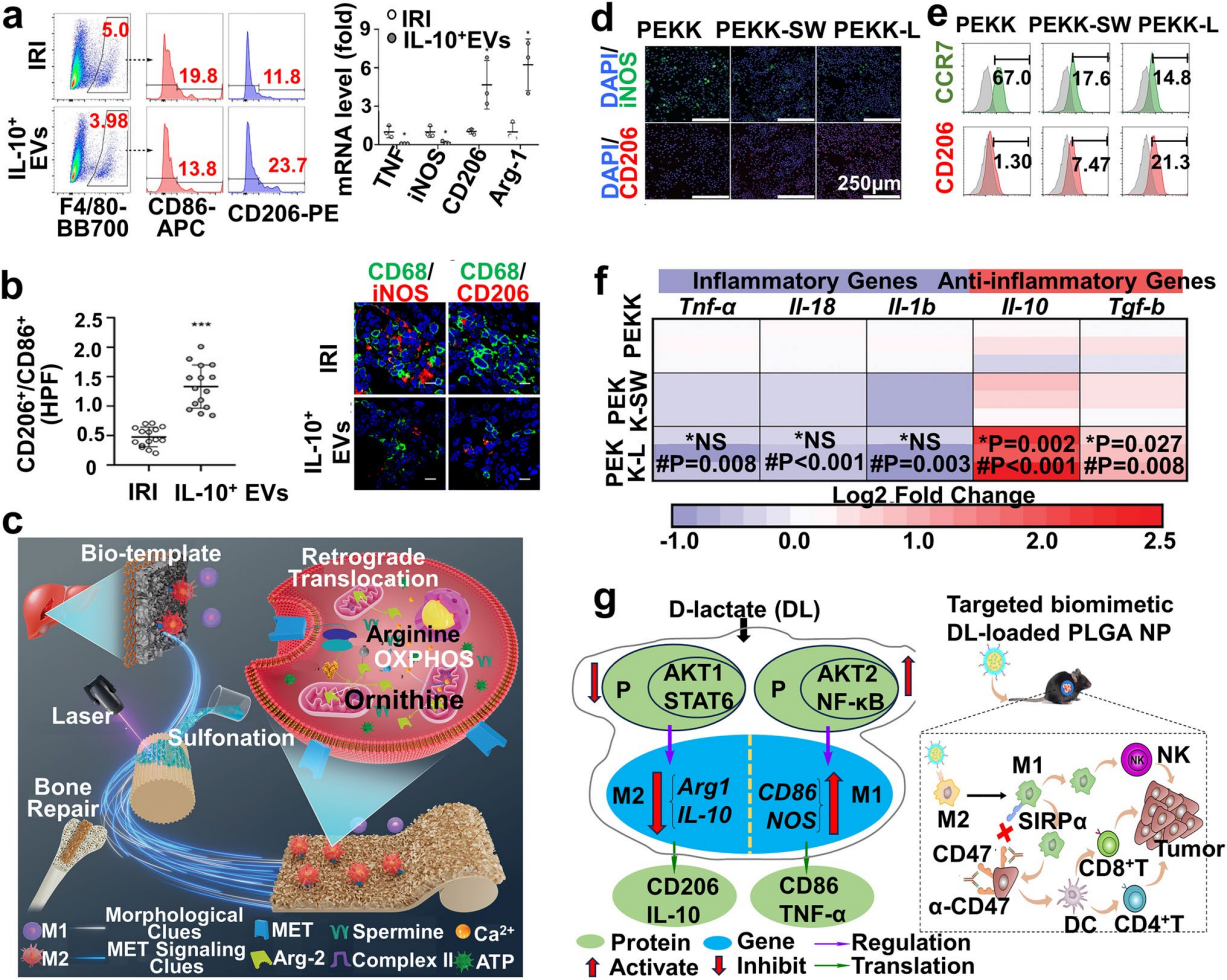
**a, b** IL-10^+^ EVs promote macrophage M2 polarization [184]. Copyright 2020, American Association for the Advancement of Science. **c** Schematic of liver-inspired PEKK scaffolds that reprogram macrophage metabolism for increasing osteoporotic osseointegration. **d, e** IF staining and biomarker (CCR7 and CD206) expression by flow cytometry of RAW 264.7 cells cultured on various scaffolds. **f** A heatmap describing the fold-change in expressed polarization genes after treatment with different groups [190]. Copyright 2023, Wiley–VCH GmbH. **g** Possible mechanisms of DL-modulated TAM repolarization from M2 to M1 (left) and remodeling of the iTME for HCC therapy via DL delivery combined with α-CD47 (right) [195]. Copyright 2023, American Association for the Advancement of Science

Exogenous IL-10 supply blocks macrophage inflammatory cascade reaction to drive M2 polarization, exhibiting enormous bioregenerative potential [272, 6]. In line with IL-4, researchers are attempting to develop an IL-10-loaded nanoplatform, which releases IL-10 via physical or chemical stimuli-triggered mechanisms, paving the way for targeted delivery [183]. Extracellular vesicles (EVs) have attracted increasing attention as feasible drug delivery systems [273]. Tang et al. [184] designed an IL-10-packaged nanoplatform (IL-10^+^ EVs) with a diameter of 134 nm by engineering dexamethasone-stimulated plasmid transfected-RAW 264.7 cells to avoid IL-10 degradation and target to the kidney. IL-10^+^ EVs favored the M2 phenotype of LPS-induced BMDMs by inactivating mTOR1, followed by its downstream targets (e.g., S6K, S6), as detected by flow cytometry, RT-qPCR, and HC, and promoted mitophagy to maintain mitochondrial homeostasis against ischemic acute kidney injury (AKI) (Fig. 7a, b). The versatile delivery strategy enabled IL-10 to evade phagocytosis, prolong circulation time, and decrease immunogenicity.

Overall, IL-4/10 link macrophages metabolism to their anti-inflammatory functionality. The major barriers, such as susceptibility to degradation and short circulation half-life, prevent IL-4/10 from being directly employed for diseases therapy. Physical encapsulation can effectively maintain the biological activity of IL-4/10, but hardly avoids their uncontrolled release. Conversely, chemical stimuli-triggered release may better satisfy the on-demand release needs while reducing off-target effect-induced side effects [274]. Current strategies have resolved pharmacokinetic challenges to some extent. However, bridging them with natural materials to exploit delivery platforms with high load efficiency, which can maintain their effective biological activity, as well as accurately orient exogenous IL-4/10 in targetable sites to achieve long-term continuous release, remains a major challenge that requires more extensive exploration.

### Amino Acid Metabolism

Metabolic adaptation in macrophages facilitates specific immunophenotype transformation [14, 275]. Among them, alterations in the metabolism of some amino acids are the earliest and foremost metabolic features that occur during macrophage polarization and define macrophage subtypes [276].

#### Arginine

Arginine metabolism remodels macrophage phenotype via the iNOS/Arg-1 axis. M(IL-4)-derived mice upregulate Arg-1 activity, whereas similar results were not observed in human macrophages. Transcription factor Fra-1 reemerges M1 macrophages by silencing Arg-1 to enhance inflammation [277]. Arg-2, another enzyme involved in L-arginine metabolism, modulates mitochondrial dynamics to upregulate OXPHOS, polarizing M1 macrophages to M2 phenotype [278, 279]. Jiang et al. [190] engineered a liver-inspired polyetherketoneketone (PEKK) scaffold with a thickness of 30 μm by femtosecond laser etching technology and sulfonation reaction. They reemerged hepatocyte growth factor receptor (MET) signaling in LPS-induced CVX-BMDMs/RAW 264.7 cells, followed by activating downstream Ras pathway, which promoted the retrograde transfer of Arg-2 from mitochondria into the cytoplasm and remodeled energy/arginine metabolism by enhancing OXPHOS to mobilize M2 phenotype for improving rat osteoporotic bone defect (demonstrated by flow cytometry, IF, ELISA, and RT-qPCR) (Fig. 7c–f). The strategic application of Arg-2 in this study verified its potential as an anti-inflammatory reserve to resurge M2 macrophages. The effect of PEKK on the isoenzyme Arg-1 has not been thoroughly studied, and it may inevitably lead to off-target effects on Arg-1. Nevertheless, there are contradictory viewpoint that Arg-2 stimulates the M1 response through mtROS and is responsible for inflammatory diseases.

#### Other Amino Acids

The regulatory role and metabolic fate of citrulline remain unclear, although citrulline can be metabolized into argininosuccinate (an inflammatory mediator) by the citrulline–NO cycle. Citrulline rapidly declines in LPS(+ IFN-γ)-primed BMDMs, and ASS1-mediated citrulline depletion is responsible for JAK2–STAT-1 signaling activation and proinflammatory cytokine generation, stimulating the M1 phenotype [280].

Carbon tracing indicates that one-third of the carbon in the TCA cycle metabolites of M2 macrophages derives from glutamine, which fuels the immune system [281], and serves as an anti-inflammatory metabolite to maintain M2 differentiation through the uridine diphosphate *N*-acetylglucosamine (UDP–GlcNAc) pathway and glutaminolysis-derived α-KG [16, 281, 282]. Glutaminolysis-derived α-KG activates M2 macrophages through Jmjd3-dependent metabolic and epigenetic reprogramming and inactivates the M1 phenotype by silencing the NF-κB pathway via PHD-regulated proline hydroxylation of protein kinase IKKβ [283]. However, glutamine anaplerosis partially replenishes succinate to activate M1 macrophages [198, 282]. Recently, dietary glutamine supply in limb ischemia-bearing diabetic mice reduced the M1/M2 ratio to promote muscle regeneration [284]. Glutaminolysis-induced M2 polarization was identified as an underlying target for obesity and type2 diabetes [282]. Contrarily, blocking glutamine metabolism skewed the macrophage phenotype toward M1 to exert antitumor immunity [194, 285]. Li et al. [189] loaded PVP-coated AuNPs into MOFs to synthesize the metabolic modulator Au@ZIF-8 via a two-step method, which enhanced GS activity (detected by a GS kit) to upregulate glutamine, altered other metabolic pathways (e.g., the TCA cycle), and remarkably decreased TNF-α secretion (analyzed by RT-qPCR) in LPS-induced RAW 264.7 cells, and applied it to glutamine metabolism-mediated diseases.

The fate of serine metabolism on macrophage polarization was investigated and a novel mechanism was proposed by which serine deficiency stimulates the M1 phenotype and inhibits STAT6-mediated M2 polarization by activating p38-dependent JAK–STAT-1 axis [286], suggesting that serine metabolism may be an underlying target. A recent study showed that serine supports LPS-dependent IL-1β production by synthesizing GSH in BMDMs and improving survival in a sepsis model by inhibiting de novo serine synthesis [287].

Considering the newly identified role of amino acid metabolism in macrophage polarization, we are optimistic that exploiting NMs to regulate amino acid metabolism within macrophages to emerge the desired phenotype will be a new research direction, although the blueprint is premature.

### Lactate Metabolism

The metabolic adaptation of M1 macrophages leads to Warburg-type lactate accumulation [49]. Emerging evidence indicates that lactate serves as an immunomodulator [288] and mediates differentially expressed gene production [289], revealing that lactate may be the white swan of reshaping energy metabolism rather than a glycolytic “waste product.” Metabolites derived from nonimmune cells have been confirmed to affect the glycolysis–OXPHOS axis of immune cells and then reshape their phenotype [290]. Tumor cell-derived lactate induces TAMs M2-related gene profiling in an HIF-1α-dependent manner for promoting tumorigenesis [291–293]. This is somewhat confusing, and the accurate mechanism remains unclear, as the conventional view is that glycolysis with lactate accumulation supports M1, whereas OXPHOS responds to M2. A recent study by Zhao may explain the contradictory paradigm, which revealed that accumulated lactate in LPS(+ IFN-γ)-driven BMDMs serves as a precursor to stimulate histone lactylation at lysine residues (known as “Kla”). Increased histone Kla activates M2-like gene transcription via epigenetic modification [294].

Currently, the two conflicting methods of supplying D-lactate (DL) and depleting lactate manipulate macrophages toward the M1 phenotype for tumor immunotherapy. DL serves as an endogenous immunoregulator that enhances the phagocytic ability of Kupffer cells (a sign of M1 macrophages) [295]. Guo et al. [195] engineered biomimetic DL-loaded PLGA NPs and decorated them with a M2-binding peptide (M2pep)-modified hepatocellular carcinoma (HCC) membrane to prepare DL@NP-M-M2pep NPs (particle size ~ 120 nm) using an extruder. These NPs accumulated in tumors through HCC membrane-related homing ability and subsequently delivered DL into IL-4-induced BMDMs via the M2 peptide-targeting capacity. This delivery drove M2-to-M1 transition, as measured via RT-qPCR, IF, and flow cytometry, thus remodeling the iTME primarily by blocking PI3K/Akt signaling downstream and activating the NF-κB pathway for carcinogen-induced HCC mice therapy (Fig. 7g). The finding first confirms the regulatory function of DL on TAM polarization and provides a reference for following research in this field.

Depleting lactate through a compatible matrix-laden lactate oxidase (Lox) [296] or bacteria has become an alternative strategy against tumor [197, 297, 298]. Regarding Lox, Liu et al. [196] loaded Lox, syrosingopine, and Gd/CeO_2_ into ZIF-8 to develop urchin-like catalysts with a size of 300 nm via a wet-chemical approach, effectively depleting intratumoral lactate by releasing Lox (detected by hippocampal analyzer and lactate kit), achieving glycometabolic reprogramming to activate the M1 response in IL-4-induced BMDMs for achieving HCC-bearing mouse immunotherapy. M1/M2 polarization was identified by RT-qPCR, IF, and ELISA. This metabolic remodeling strategy targeting lactate may open a promising avenue for tumor therapy, which can be expanded to other representative metabolites in the iTME. Conversely, our group [299] incorporated engineered *Lactococcus lactis* (*L. lactis*) into a heparin-poloxamer copolymer to prepare a living hydrogel delivery system (HP@LL_VEGF), which steadily secreted lactate from *L. lactis* as a metabolic signal to remodel LPS + IFN-γ-induced BMDMs toward M2-like, (confirmed by IF, CLSM, flow cytometry, RT-qPCR, and WB, thus ameliorating inflammation in streptozotocin-induced diabetic mice and accelerating wound healing.

Lactate consumption and DL replenishment are believed to support macrophage M2-to-M1 polarization against tumor growth. However, these two mainstream strategies are contradictory, and researchers have not accurately elucidated the regulatory mechanism by which designed NMs influence macrophage polarization by regulating lactate levels. Lactate serves not only as a byproduct of glycolysis but also as a factor driving M2 polarization via Kla-induced Arg-1 expression. Addressing how to selectively deplete or supply lactate to avoid side effects on other physiological cells or tissues may be a major issue for researchers to consider.

## Conclusion and Perspectives

NM-based repurposing of macrophage metabolism holds considerable potential in various disease scenario. Here, we outline recent advances in the application of NM-based metabolic reprogramming, providing a partial blueprint and insights into macrophage metabolic immunotherapy. We believe that a deeper understanding of the interaction between metabolism, polarization, and NMs can greatly accelerate the exploration of next-generation NMs with optimal performance under the strategic collaboration of multiple disciplines. Nevertheless, thorough research is needed in the future to conquer remaining challenges that have not been addressed and bring engineered NMs into clinical applications. The following section introduces additional information on the key issues that need to be addressed and future research priorities, yielding insights for future research.

1. Engineering NMs with macrophage-targeting moieties (e.g., ligand) to enhance phagocytic uptake or bridging stimuli-triggered units may achieve personalized targeting [300, 301]. NMs confront a series of pathophysiologic barriers during transport in vivo. Their complex surface interfaces inevitably interact with proteins and cells in blood and tissues, leading to performance change and inevitable clearance by the immune system [300]. NMs are inevitably internalized by other macrophage subsets or adjacent cells even within the same tissue due to the ubiquitous distribution of cells, resulting in unintended off-target effects and immunogenicity. Establishing a biomimetic Trojan delivery system that accurately targets specific macrophages subsets remains a challenge before entering clinical research. The pathogenesis of metabolic diseases is extremely complex, involving multiple altered parameters. Restricted by suboptimal efficacy of a single target and limited target screening [302], the perspective should gradually shift from the doctrine of “one molecule, one target, one disease” to “all-in-one” NMs based on a multipronged strategy that synergistically modulates multiple metabolic targets in macrophages [175].
2. The exploration of theranostic NMs requires more rigorous consideration to devise programmable cargo transport strategies, demonstrating a diagnostic logic to determine cargo release time, followed by a therapeutic logic to program cargo release, to timely adjust the therapeutic schedule. More metabolic parameters, such as fumarate, α-KG, GLUT1, glycolytic enzymes (e.g., PKM2, hexokinase, pyruvate dehydrogenase), FAS-associated enzymes (e.g., fatty acid synthase, acetyl-CoA carboxylase), and other amino acids (e.g., tryptophan, methionine), also undergo significant alteration in the metabolic adaptation of macrophages [24, 303–306] and can serve as theoretical targets for innovative therapies. These are beyond the scope of this review and have not been extensively explored yet. A combination of imaging technologies and metabolic events has enabled us to visualize and quantify macrophage behaviors. To accelerate clinical translation, clinically mature imaging techniques (e.g., MRI, CT) and FDA-approved materials (e.g., PEG) should be prioritized to exploit nanomedicines.
3. Most preclinical results originate from cell lines and animal models, and not humans. Metabolic profiles vary between them and humans, as is the behavior of NMs [307, 308]. Undoubtedly, human tissue models will be introduced into metabolic research to eliminate the long-standing dependence on traditional models [309], such as 3D organoids, which better mimic human organs [310]. Due to inappropriate assessment models, inadequate understanding of nano–bio-interactions, etc., current macrophage nanomedicines rarely achieve practical clinical translation [311], as evidenced by their limited commercialization. Long-term safe administration in humans is essential. The biodegradability, pharmacokinetics, and long-term toxicity of NMs must be analyzed in detail. Perhaps we can explore intracellular behavior of internalized NMs utilizing computational toxicology, micro “organ on chip” devices, and high-throughput drug screening to establish pharmacokinetic–pharmacodynamic models for elucidating the toxicological pathway and therapeutic value. The minimum information reporting in bio–nano-experimental literature (MIRIBEL) standard should be followed for accelerating their clinical translation [312].
4. Precision nanomedicine may be the future research trend, aimed to tailor patient-personalized therapeutic regimens through various cutting-edge technologies based on individual differences, to minimize iatrogenic damage and medical costs and maximize patient benefits [313]. This strategy probably avoids an adverse drug response caused by a “one size fits all” treatment without considering patient heterogeneity [314]. Similar variability probably exists in simple NMs labeled with targeted fragments. Algorithms (e.g., artificial intelligence) may be imperative for implementing precision medicine, which contribute to patient classification and NM design, such as predicting optimal size, pharmacokinetics, and prescreening NMs for better patient compliance [315]. Some questions are raised frequently about precision medicine: Can we provide tailor-designed nanomedicines for each patient? Besides the complex clinical approval process of personalized nanomedicine customization, the limitations of its manufacturing technology and high-cost development are questions for the future.

Overall, although these strategies highlight the impressive preclinical potential of NM-based macrophage immunotherapy, very few have directly entered the clinical arena. This recognition has prompted some experts to raise provocative questions, hoping to transform the discipline from merely potential platforms to those enabling to provide positive clinical outcomes. Encouraged by the prominent progress already made, it could be expected that macrophage immuno-nanomedicines will eventually enter the clinic and market in the near future.

## Statistical Analysis

Each experiment in the cited literature was independently repeated at least three times, and all quantitative data were expressed as mean ± standard deviation (X ± SD). The sample size for all experiments met the requirements for statistical analysis. Pairwise comparisons of consecutive variables were analyzed using a two-tailed Student’s *t* test to determine statistical significance for normally distributed variables. Non-normally distributed variables were analyzed using nonparametric tests such as the Mann–Whitney U test (Wilcoxon rank sum test) or the Kruskal–Wallis H test followed by Dunn’s multiple-comparison test. The chi-square test was employed to compare and analyze the statistical significance of categorical variables between two groups. For multiple comparisons of variables, one-way ANOVA followed by Tukey’s post hoc test was conducted, and two-way ANOVA was used for analyses involving two independent variables. Statistical analyses were performed using GraphPad Prism 9.5 software, with a *P* value of < 0.05 considered statistically significant. The confidence interval for all statistical tests was set at 95%.

With the help of multiple databases, we identified potential biomarkers of metabolic pathways using Progenesis QI software (Waters Corporation, Milford, USA) for metabolomics analysis. These databases include the Human Metabolome Database (HMDB, http://hmdb.ca/), Genomes (KEGG, http://www.genome.jp/kegg/), mzCloud (https://www.mzcloud.org/), and MBRole 2.0 (http://csbg.cnb.csic.es/mbrole2/). The metabolites were screened using a combination of multidimensional and one-dimensional analysis methods.

## References

[1] K. Sadtler, A. Singh, M.T. Wolf, X. Wang, D.M. Pardoll et al., Design, clinical translation and immunological response of biomaterials in regenerative medicine. Nat. Rev. Mater. **1**, 16040–16056 (2016). 10.1038/natrevmats.2016.40

[2] R. Sridharan, A.R. Cameron, D.J. Kelly, C.J. Kearney, F.J. O’Brien, Biomaterial based modulation of macrophage polarization: a review and suggested design principles. Mater. Today **18**(6), 313–325 (2015). 10.1016/j.mattod.2015.01.019

[3] T.A. Wynn, A. Chawla, J.W. Pollard, Macrophage biology in development, homeostasis and disease. Nature **496**(7446), 445–455 (2013). 10.1038/nature12034 23619691 10.1038/nature12034 PMC3725458

[4] L. Parisi, E. Gini, D. Baci, M. Tremolati, M. Fanuli et al., Macrophage polarization in chronic inflammatory diseases: Killers or builders? J. Immunol. Res. **2018**, 8917804 (2018). 10.1155/2018/8917804 29507865 10.1155/2018/8917804 PMC5821995

[5] D.M. Mosser, J.P. Edwards, Exploring the full spectrum of macrophage activation. Nat. Rev. Immunol. **8**(12), 958–969 (2008). 10.1038/nri2448 19029990 10.1038/nri2448 PMC2724991

[6] A. Mantovani, A. Sica, S. Sozzani, P. Allavena, A. Vecchi et al., The chemokine system in diverse forms of macrophage activation and polarization. Trends Immunol. **25**(12), 677–686 (2004). 10.1016/j.it.2004.09.015 15530839 10.1016/j.it.2004.09.015

[7] J.J. O’Shea, P.J. Murray, Cytokine signaling modules in inflammatory responses. Immunity **28**(4), 477–487 (2008). 10.1016/j.immuni.2008.03.002 18400190 10.1016/j.immuni.2008.03.002 PMC2782488

[8] A. Shapouri-Moghaddam, S. Mohammadian, H. Vazini, M. Taghadosi, S.A. Esmaeili et al., Macrophage plasticity, polarization, and function in health and disease. J. Cell. Physiol. **233**(9), 6425–6440 (2018). 10.1002/jcp.26429 10.1002/jcp.26429 PMC13482560 29319160

[9] S. Gordon, F.O. Martinez, Alternative activation of macrophages: mechanism and functions. Immunity **32**(5), 593–604 (2010). 10.1016/j.immuni.2010.05.007 20510870 10.1016/j.immuni.2010.05.007

[10] W. Xu, X. Zhao, M.R. Daha, C. van Kooten, Reversible differentiation of pro- and anti-inflammatory macrophages. Mol. Immunol. **53**(3), 179–186 (2013). 10.1016/j.molimm.2012.07.005 22944456 10.1016/j.molimm.2012.07.005

[11] E.L. Mills, B. Kelly, A. Logan, A.S.H. Costa, M. Varma et al., Succinate dehydrogenase supports metabolic repurposing of mitochondria to drive inflammatory macrophages. Cell **167**(2), 457-470.e413 (2016). 10.1016/j.cell.2016.08.064 27667687 10.1016/j.cell.2016.08.064 PMC5863951

[12] D.G. Ryan, L.A.J. O’Neill, Krebs cycle reborn in macrophage immunometabolism. Annu. Rev. Immunol. **38**, 289–313 (2020). 10.1146/annurev-immunol-081619-104850 31986069 10.1146/annurev-immunol-081619-104850

[13] E.L. Mills, B. Kelly, L.A.J. O’Neill, Mitochondria are the powerhouses of immunity. Nat. Immunol. **18**(5), 488–498 (2017). 10.1038/ni.3704 28418387 10.1038/ni.3704

[14] J. Van den Bossche, L.A. O’Neill, D. Menon, Macrophage immunometabolism: where are we (going)? Trends Immunol. **38**(6), 395–406 (2017). 10.1016/j.it.2017.03.001 28396078 10.1016/j.it.2017.03.001

[15] E.L. Mills, L.A. O’Neill, Reprogramming mitochondrial metabolism in macrophages as an anti-inflammatory signal. Eur. J. Immunol. **46**(1), 13–21 (2016). 10.1002/eji.201445427 26643360 10.1002/eji.201445427

[16] A.K. Jha, S.C. Huang, A. Sergushichev, V. Lampropoulou, Y. Ivanova et al., Network integration of parallel metabolic and transcriptional data reveals metabolic modules that regulate macrophage polarization. Immunity **42**(3), 419–430 (2015). 10.1016/j.immuni.2015.02.005 25786174 10.1016/j.immuni.2015.02.005

[17] A.M. Kabat, E.J. Pearce, Inflammation by way of macrophage metabolism. Science **356**(6337), 488–489 (2017). 10.1126/science.aan2691 28473549 10.1126/science.aan2691

[18] I. Vitale, G. Manic, L.M. Coussens, G. Kroemer, L. Galluzzi, Macrophages and metabolism in the tumor microenvironment. Cell Metab. **30**(1), 36–50 (2019). 10.1016/j.cmet.2019.06.001 31269428 10.1016/j.cmet.2019.06.001

[19] J. Li, X. Jiang, H. Li, M. Gelinsky, Z. Gu, Tailoring materials for modulation of macrophage fate. Adv. Mater. **33**(12), e2004172 (2021). 10.1002/adma.202004172 33565154 10.1002/adma.202004172 PMC9245340

[20] L. Zhang, X. Chen, P. Cai, H. Sun, S. Shen et al., Reprogramming mitochondrial metabolism in synovial macrophages of early osteoarthritis by a camouflaged meta-defensome. Adv. Mater. **34**(30), e2202715 (2022). 10.1002/adma.202202715 35671349 10.1002/adma.202202715

[21] L. Wang, D. Wang, T. Zhang, Y. Ma, X. Tong et al., The role of immunometabolism in macrophage polarization and its impact on acute lung injury/acute respiratory distress syndrome. Front. Immunol. **14**, 1117548 (2023). 10.3389/fimmu.2023.1117548 37020557 10.3389/fimmu.2023.1117548 PMC10067752

[22] S. Cai, M. Zhao, B. Zhou, A. Yoshii, D. Bugg et al., Mitochondrial dysfunction in macrophages promotes inflammation and suppresses repair after myocardial infarction. J. Clin. Invest. **133**(4), e159498 (2023). 10.1172/JCI159498 36480284 10.1172/JCI159498 PMC9927948

[23] S.A. Eming, P.J. Murray, E.J. Pearce, Metabolic orchestration of the wound healing response. Cell Metab. **33**(9), 1726–1743 (2021). 10.1016/j.cmet.2021.07.017 34384520 10.1016/j.cmet.2021.07.017

[24] A. Castegna, R. Gissi, A. Menga, M. Montopoli, M. Favia et al., Pharmacological targets of metabolism in disease: opportunities from macrophages. Pharmacol. Ther. **210**, 107521 (2020). 10.1016/j.pharmthera.2020.107521 32151665 10.1016/j.pharmthera.2020.107521

[25] P. Hou, J. Fang, Z. Liu, Y. Shi, M. Agostini et al., Macrophage polarization and metabolism in atherosclerosis. Cell Death Dis. **14**(10), 691 (2023). 10.1038/s41419-023-06206-z 37863894 10.1038/s41419-023-06206-z PMC10589261

[26] W.K.E. Ip, N. Hoshi, D.S. Shouval, S. Snapper, R. Medzhitov, Anti-inflammatory effect of IL-10 mediated by metabolic reprogramming of macrophages. Science **356**(6337), 513–519 (2017). 10.1126/science.aal3535 28473584 10.1126/science.aal3535 PMC6260791

[27] R.A. Franklin, W. Liao, A. Sarkar, M.V. Kim, M.R. Bivona et al., The cellular and molecular origin of tumor-associated macrophages. Science **344**(6186), 921–925 (2014). 10.1126/science.1252510 24812208 10.1126/science.1252510 PMC4204732

[28] M. Li, Y. Yang, L. Xiong, P. Jiang, J. Wang et al., Metabolism, metabolites, and macrophages in cancer. J. Hematol. Oncol. **16**(1), 80 (2023). 10.1186/s13045-023-01478-6 37491279 10.1186/s13045-023-01478-6 PMC10367370

[29] D. Kim, Y.W. Wu, Q.Y. Li, Y.K. Oh, Nanoparticle-mediated lipid metabolic reprogramming of T cells in tumor microenvironments for immunometabolic therapy. Nano-Micro Lett. **13**(1), 31 (2021). 10.1007/s40820-020-00555-6 10.1007/s40820-020-00555-6 PMC8006499 34138236

[30] T. Hou, T. Wang, W. Mu, R. Yang, S. Liang et al., Nanoparticle-loaded polarized-macrophages for enhanced tumor targeting and cell-chemotherapy. Nano-Micro Lett. **13**(1), 6 (2020). 10.1007/s40820-020-00531-0 10.1007/s40820-020-00531-0 PMC8187668 34138195

[31] J. Mao, L. Chen, Z. Cai, S. Qian, Z. Liu et al., Advanced biomaterials for regulating polarization of macrophages in wound healing. Adv. Funct. Mater. **32**(12), 2111003 (2021). 10.1002/adfm.202111003

[32] E. Peled, A. Sosnik, Amphiphilic galactomannan nanoparticles trigger the alternative activation of murine macrophages. J. Control. Release **339**, 473–483 (2021). 10.1016/j.jconrel.2021.10.017 34662585 10.1016/j.jconrel.2021.10.017

[33] J. Tang, S. Baxter, A. Menon, A. Alaarg, B.L. Sanchez-Gaytan et al., Immune cell screening of a nanoparticle library improves atherosclerosis therapy. Proc. Natl. Acad. Sci. U.S.A. **113**(44), E6731–E6740 (2016). 10.1073/pnas.1609629113 27791119 10.1073/pnas.1609629113 PMC5098679

[34] H.J. Jeong, R.J. Yoo, J.K. Kim, M.H. Kim, S.H. Park et al., Macrophage cell tracking pet imaging using mesoporous silica nanoparticles via in vivo bioorthogonal F-18 labeling. Biomaterials **199**, 32–39 (2019). 10.1016/j.biomaterials.2019.01.043 30735894 10.1016/j.biomaterials.2019.01.043

[35] S.B. Lee, H.W. Lee, T.D. Singh, Y. Li, S.K. Kim et al., Visualization of macrophage recruitment to inflammation lesions using highly sensitive and stable radionuclide-embedded gold nanoparticles as a nuclear bio-imaging platform. Theranostics **7**(4), 926–934 (2017). 10.7150/thno.17131 28382164 10.7150/thno.17131 PMC5381254

[36] J. Toczek, P. Boodagh, N. Sanzida, M. Ghim, M. Salarian et al., Computed tomography imaging of macrophage phagocytic activity in abdominal aortic aneurysm. Theranostics **11**(12), 5876–5888 (2021). 10.7150/thno.55106 33897887 10.7150/thno.55106 PMC8058712

[37] Z. Guo, L. Yang, M. Chen, X. Wen, H. Liu et al., Molecular imaging of advanced atherosclerotic plaques with folate receptor-targeted 2D nanoprobes. Nano Res. **13**(1), 173–182 (2019). 10.1007/s12274-019-2592-4

[38] H. Deng, X. Li, J. Ju, X. Mo, G. Ge et al., Multifunctional nanoprobes for macrophage imaging. Biomaterials **290**, 121824 (2022). 10.1016/j.biomaterials.2022.121824 36209580 10.1016/j.biomaterials.2022.121824

[39] A. Ramesh, N. Deshpande, V. Malik, A. Nguyen, M. Malhotra et al., Activatable nanoreporters for real-time tracking of macrophage phenotypic states associated with disease progression. Small **19**(41), e2300978 (2023). 10.1002/smll.202300978 37317008 10.1002/smll.202300978

[40] X. Liu, X. Xie, J. Jiang, M. Lin, E. Zheng et al., Use of nanoformulation to target macrophages for disease treatment. Adv. Funct. Mater. **31**(38), 2104487 (2021). 10.1002/adfm.202104487

[41] Y. Zhu, S. Hideyoshi, H. Jiang, Y. Matsumura, J.L. Dziki et al., Injectable, porous, biohybrid hydrogels incorporating decellularized tissue components for soft tissue applications. Acta Biomater. **73**, 112–126 (2018). 10.1016/j.actbio.2018.04.003 29649634 10.1016/j.actbio.2018.04.003 PMC5985206

[42] H. Yao, F. Wang, H. Chong, J. Wang, Y. Bai et al., A curcumin-modified coordination polymers with ROS scavenging and macrophage phenotype regulating properties for efficient ulcerative colitis treatment. Adv. Sci. **10**(19), e2300601 (2023). 10.1002/advs.202300601 10.1002/advs.202300601 PMC10323614 37195012

[43] L. Hou, X. Gong, J. Yang, H. Zhang, W. Yang et al., Hybrid-membrane-decorated prussian blue for effective cancer immunotherapy via tumor-associated macrophages polarization and hypoxia relief. Adv. Mater. **34**(14), e2200389 (2022). 10.1002/adma.202200389 35103352 10.1002/adma.202200389

[44] P.K. Langston, M. Shibata, T. Horng, Metabolism supports macrophage activation. Front. Immunol. **8**, 61 (2017). 10.3389/fimmu.2017.00061 28197151 10.3389/fimmu.2017.00061 PMC5281575

[45] D.E. Lackey, J.M. Olefsky, Regulation of metabolism by the innate immune system. Nat. Rev. Endocrinol. **12**(1), 15–28 (2016). 10.1038/nrendo.2015.189 26553134 10.1038/nrendo.2015.189

[46] O. Osborn, J.M. Olefsky, The cellular and signaling networks linking the immune system and metabolism in disease. Nat. Med. **18**(3), 363–374 (2012). 10.1038/nm.2627 22395709 10.1038/nm.2627

[47] G.J. Koelwyn, E.M. Corr, E. Erbay, K.J. Moore, Regulation of macrophage immunometabolism in atherosclerosis. Nat. Immunol. **19**(6), 526–537 (2018). 10.1038/s41590-018-0113-3 29777212 10.1038/s41590-018-0113-3 PMC6314674

[48] V. Kumar, Targeting macrophage immunometabolism: dawn in the darkness of sepsis. Int. Immunopharmacol. **58**, 173–185 (2018). 10.1016/j.intimp.2018.03.005 29625385 10.1016/j.intimp.2018.03.005

[49] S. Galvan-Pena, L.A. O’Neill, Metabolic reprograming in macrophage polarization. Front. Immunol. **5**, 420 (2014). 10.3389/fimmu.2014.00420 25228902 10.3389/fimmu.2014.00420 PMC4151090

[50] M. Xie, Y. Yu, R. Kang, S. Zhu, L. Yang et al., PKM2-dependent glycolysis promotes NLRP3 and AIM2 inflammasome activation. Nat. Commun. **7**, 13280 (2016). 10.1038/ncomms13280 27779186 10.1038/ncomms13280 PMC5093342

[51] E.M. Palsson-McDermott, A.M. Curtis, G. Goel, M.A.R. Lauterbach, F.J. Sheedy et al., Pyruvate kinase M2 regulates Hif-1alpha activity and IL-1beta induction and is a critical determinant of the warburg effect in LPS-activated macrophages. Cell Metab. **21**(2), 347 (2015). 10.1016/j.cmet.2015.01.017 29510100 10.1016/j.cmet.2015.01.017

[52] K. Mehla, P.K. Singh, Metabolic regulation of macrophage polarization in cancer. Trends Cancer **5**(12), 822–834 (2019). 10.1016/j.trecan.2019.10.007 31813459 10.1016/j.trecan.2019.10.007 PMC7187927

[53] L.A. O’Neill, R.J. Kishton, J. Rathmell, A guide to immunometabolism for immunologists. Nat. Rev. Immunol. **16**(9), 553–565 (2016). 10.1038/nri.2016.70 27396447 10.1038/nri.2016.70 PMC5001910

[54] B.J. Altman, Z.E. Stine, C.V. Dang, From krebs to clinic: glutamine metabolism to cancer therapy. Nat. Rev. Cancer **16**(11), 749 (2016). 10.1038/nrc.2016.114 28704361 10.1038/nrc.2016.114

[55] M. Trauelsen, T.K. Hiron, D. Lin, J.E. Petersen, B. Breton et al., Extracellular succinate hyperpolarizes M2 macrophages through SUCNR1/GRP91-mediated gq signaling. Cell Rep. **35**(11), 109246 (2021). 10.1016/j.celrep.2021.109246 34133934 10.1016/j.celrep.2021.109246

[56] M. de Castro Fonseca, C.J. Aguiar, J.A. da Rocha Franco, R.N. Gingold, M.F. Leite, GRP91: expanding the frontiers of Krebs cycle intermediates. Cell Commun. Signal **14**, 3 (2016). 10.1186/s12964-016-0126-1 26759054 10.1186/s12964-016-0126-1 PMC4709936

[57] T. Reimann, D. Buscher, R.A. Hipskind, S. Krautwald, M.L. Lohmann-Matthes et al., Lipopolysaccharide induces activation of the Raf-1/MAP kinase pathway. A putative role for Raf-1 in the induction of the IL-1 beta and the TNF-alpha genes. J. Immunol. **153**(12), 5740–5749 (1994). 10.4049/jimmunol.153.12.5740 7989771

[58] M. Rath, I. Muller, P. Kropf, E.I. Closs, M. Munder, Metabolism via arginase or nitric oxide synthase: two competing arginine pathways in macrophages. Front. Immunol. **5**, 532 (2014). 10.3389/fimmu.2014.00532 25386178 10.3389/fimmu.2014.00532 PMC4209874

[59] J.E. Qualls, C. Subramanian, W. Rafi, A.M. Smith, L. Balouzian et al., Sustained generation of nitric oxide and control of mycobacterial infection requires argininosuccinate synthase 1. Cell Host Microbe **12**(3), 313–323 (2012). 10.1016/j.chom.2012.07.012 22980328 10.1016/j.chom.2012.07.012 PMC3444824

[60] B. Beltran, A. Mathur, M.R. Duchen, J.D. Erusalimsky, S. Moncada, The effect of nitric oxide on cell respiration: a key to understanding its role in cell survival or death. Proc. Natl. Acad. Sci. U.S.A. **97**(26), 14602–14607 (2000). 10.1073/pnas.97.26.14602 11121062 10.1073/pnas.97.26.14602 PMC18965

[61] J. Van den Bossche, J. Baardman, N.A. Otto, S. van der Velden, A.E. Neele et al., Mitochondrial dysfunction prevents repolarization of inflammatory macrophages. Cell Rep. **17**(3), 684–696 (2016). 10.1016/j.celrep.2016.09.008 27732846 10.1016/j.celrep.2016.09.008

[62] E.L. Pearce, E.J. Pearce, Metabolic pathways in immune cell activation and quiescence. Immunity **38**(4), 633–643 (2013). 10.1016/j.immuni.2013.04.005 23601682 10.1016/j.immuni.2013.04.005 PMC3654249

[63] M. Hesse, M. Modolell, A.C. La Flamme, M. Schito, J.M. Fuentes et al., Differential regulation of nitric oxide synthase-2 and arginase-1 by type 1/type 2 cytokines in vivo: granulomatous pathology is shaped by the pattern of L-arginine metabolism. J. Immunol. **167**(11), 6533–6544 (2001). 10.4049/jimmunol.167.11.6533 11714822 10.4049/jimmunol.167.11.6533

[64] J. Van den Bossche, W.H. Lamers, E.S. Koehler, J.M. Geuns, L. Alhonen et al., Pivotal advance: arginase-1-independent polyamine production stimulates the expression of IL-4-induced alternatively activated macrophage markers while inhibiting LPS-induced expression of inflammatory genes. J. Leukoc. Biol. **91**(5), 685–699 (2012). 10.1189/jlb.0911453 22416259 10.1189/jlb.0911453

[65] N. Sukhbaatar, T. Weichhart, Iron regulation: macrophages in control. Pharmaceuticals **11**(4), 137 (2018). 10.3390/ph11040137 30558109 10.3390/ph11040137 PMC6316009

[66] D.J. Kosman, Redox cycling in iron uptake, efflux, and trafficking. J. Biol. Chem. **285**(35), 26729–26735 (2010). 10.1074/jbc.R110.113217 20522542 10.1074/jbc.R110.113217 PMC2930670

[67] M.P. Soares, I. Hamza, Macrophages and iron metabolism. Immunity **44**(3), 492–504 (2016). 10.1016/j.immuni.2016.02.016 26982356 10.1016/j.immuni.2016.02.016 PMC4794998

[68] E. Nemeth, M.S. Tuttle, J. Powelson, M.B. Vaughn, A. Donovan et al., Hepcidin regulates cellular iron efflux by binding to ferroportin and inducing its internalization. Science **306**(5704), 2090–2093 (2004). 10.1126/science.1104742 15514116 10.1126/science.1104742

[69] X.B. Liu, N.B. Nguyen, K.D. Marquess, F. Yang, D.J. Haile, Regulation of hepcidin and ferroportin expression by lipopolysaccharide in splenic macrophages. Blood Cells Mol. Dis. **35**(1), 47–56 (2005). 10.1016/j.bcmd.2005.04.006 15932798 10.1016/j.bcmd.2005.04.006

[70] G. Cairo, S. Recalcati, A. Mantovani, M. Locati, Iron trafficking and metabolism in macrophages: contribution to the polarized phenotype. Trends Immunol. **32**(6), 241–247 (2011). 10.1016/j.it.2011.03.007 21514223 10.1016/j.it.2011.03.007

[71] M. Pereira, T.D. Chen, N. Buang, A. Olona, J.H. Ko et al., Acute iron deprivation reprograms human macrophage metabolism and reduces inflammation in vivo. Cell Rep. **28**(2), 498-511.e495 (2019). 10.1016/j.celrep.2019.06.039 31291584 10.1016/j.celrep.2019.06.039 PMC6635384

[72] A.B. Parekh, J.W. Putney Jr., Store-operated calcium channels. Physiol. Rev. **85**(2), 757–810 (2005). 10.1152/physrev.00057.2003 15788710 10.1152/physrev.00057.2003

[73] S. Feske, H. Wulff, E.Y. Skolnik, Ion channels in innate and adaptive immunity. Annu. Rev. Immunol. **33**, 291–353 (2015). 10.1146/annurev-immunol-032414-112212 25861976 10.1146/annurev-immunol-032414-112212 PMC4822408

[74] C. Giorgi, S. Marchi, P. Pinton, The machineries, regulation and cellular functions of mitochondrial calcium. Nat. Rev. Mol. Cell Biol. **19**(11), 713–730 (2018). 10.1038/s41580-018-0052-8 30143745 10.1038/s41580-018-0052-8

[75] P. He, F. Liu, M. Li, M. Ren, X. Wang et al., Mitochondrial calcium ion nanogluttons alleviate periodontitis via controlling mPTPs. Adv. Healthc. Mater. **12**(15), e2203106 (2023). 10.1002/adhm.202203106 36906927 10.1002/adhm.202203106

[76] O. Krenkel, F. Tacke, Liver macrophages in tissue homeostasis and disease. Nat. Rev. Immunol. **17**(5), 306–321 (2017). 10.1038/nri.2017.11 28317925 10.1038/nri.2017.11

[77] D. Vats, L. Mukundan, J.I. Odegaard, L. Zhang, K.L. Smith et al., Oxidative metabolism and PGC-1beta attenuate macrophage-mediated inflammation. Cell Metab. **4**(1), 13–24 (2006). 10.1016/j.cmet.2006.05.011 16814729 10.1016/j.cmet.2006.05.011 PMC1904486

[78] J.T. Hou, K.K. Yu, K. Sunwoo, W.Y. Kim, S. Koo et al., Fluorescent imaging of reactive oxygen and nitrogen species associated with pathophysiological processes. Chem **6**(4), 832–866 (2020). 10.1016/j.chempr.2019.12.005

[79] L.A. O’Neill, E.J. Pearce, Immunometabolism governs dendritic cell and macrophage function. J. Exp. Med. **213**(1), 15–23 (2016). 10.1084/jem.20151570 26694970 10.1084/jem.20151570 PMC4710204

[80] E. Rendra, V. Riabov, D.M. Mossel, T. Sevastyanova, M.C. Harmsen et al., Reactive oxygen species (ROS) in macrophage activation and function in diabetes. Immunobiology **224**(2), 242–253 (2019). 10.1016/j.imbio.2018.11.010 30739804 10.1016/j.imbio.2018.11.010

[81] L. Formentini, F. Santacatterina, C. Nunez de Arenas, K. Stamatakis, D. Lopez-Martinez et al., Mitochondrial ROS production protects the intestine from inflammation through functional M2 macrophage polarization. Cell Rep. **19**(6), 1202–1213 (2017). 10.1016/j.celrep.2017.04.036 28494869 10.1016/j.celrep.2017.04.036

[82] A.P. West, I.E. Brodsky, C. Rahner, D.K. Woo, H. Erdjument-Bromage et al., TLR signalling augments macrophage bactericidal activity through mitochondrial ROS. Nature **472**(7344), 476–480 (2011). 10.1038/nature09973 21525932 10.1038/nature09973 PMC3460538

[83] M. Hori, K. Nishida, Oxidative stress and left ventricular remodelling after myocardial infarction. Cardiovasc. Res. **81**(3), 457–464 (2009). 10.1093/cvr/cvn335 19047340 10.1093/cvr/cvn335

[84] X. Li, Y. Liu, X. Qi, S. Xiao, Z. Xu et al., Sensitive activatable nanoprobes for real-time ratiometric magnetic resonance imaging of reactive oxygen species and ameliorating inflammation in vivo. Adv. Mater. **34**(19), e2109004 (2022). 10.1002/adma.202109004 35276751 10.1002/adma.202109004

[85] B.X. Ma, H. Xu, W.H. Zhuang, Y.N. Wang, G.C. Li et al., Reactive oxygen species responsive theranostic nanoplatform for two-photon aggregation-induced emission imaging and therapy of acute and chronic inflammation. ACS Nano **14**(5), 5862–5873 (2020). 10.1021/acsnano.0c01012 32379416 10.1021/acsnano.0c01012

[86] H. Wang, D. Yu, B. Li, Z. Liu, J. Ren et al., Ultrasensitive magnetic resonance imaging of systemic reactive oxygen species in vivo for early diagnosis of sepsis using activatable nanoprobes. Chem. Sci. **10**(13), 3770–3778 (2019). 10.1039/c8sc04961k 30996965 10.1039/c8sc04961k PMC6447818

[87] B.A. Berkowitz, Oxidative stress measured in vivo without an exogenous contrast agent using quest MRI. J. Magn. Reson. **291**, 94–100 (2018). 10.1016/j.jmr.2018.01.013 29705036 10.1016/j.jmr.2018.01.013 PMC5963509

[88] E.M. Espinoza, J.J. Roise, I.C. Li, R. Das, N. Murthy, Advances in imaging reactive oxygen species. J. Nucl. Med. **62**(4), 457–461 (2021). 10.2967/jnumed.120.245415 33384322 10.2967/jnumed.120.245415 PMC8049370

[89] B.J. Crielaard, C.J. Rijcken, L. Quan, S. van der Wal, I. Altintas et al., Glucocorticoid-loaded core-cross-linked polymeric micelles with tailorable release kinetics for targeted therapy of rheumatoid arthritis. Angew. Chem. Int. Ed. **51**(29), 7254–7258 (2012). 10.1002/anie.201202713 10.1002/anie.201202713 PMC3499037 22692876

[90] G. Yang, M. Fan, J. Zhu, C. Ling, L. Wu et al., A multifunctional anti-inflammatory drug that can specifically target activated macrophages, massively deplete intracellular H_2_O_2_, and produce large amounts CO for a highly efficient treatment of osteoarthritis. Biomaterials **255**, 120155 (2020). 10.1016/j.biomaterials.2020.120155 32554130 10.1016/j.biomaterials.2020.120155

[91] A. Bellahcene, M.J. Nokin, V. Castronovo, C. Schalkwijk, Methylglyoxal-derived stress: an emerging biological factor involved in the onset and progression of cancer. Semin. Cancer Biol. **49**, 64–74 (2018). 10.1016/j.semcancer.2017.05.010 28600198 10.1016/j.semcancer.2017.05.010

[92] Z. Mao, M. Ye, W. Hu, X. Ye, Y. Wang et al., Design of a ratiometric two-photon probe for imaging of hypochlorous acid (HClO) in wounded tissues. Chem. Sci. **9**(28), 6035–6040 (2018). 10.1039/c8sc01697f 30079216 10.1039/c8sc01697f PMC6052737

[93] M. Yao, Y. Lu, L. Shi, Y. Huang, Q. Zhang et al., A ROS-responsive, self-immolative and self-reporting hydrogen sulfide donor with multiple biological activities for the treatment of myocardial infarction. Bioact. Mater. **9**, 168–182 (2022). 10.1016/j.bioactmat.2021.07.011 34820564 10.1016/j.bioactmat.2021.07.011 PMC8586025

[94] H. Feng, Z. Zhang, Q. Meng, H. Jia, Y. Wang et al., Rapid response fluorescence probe enabled in vivo diagnosis and assessing treatment response of hypochlorous acid-mediated rheumatoid arthritis. Adv. Sci. **5**(8), 1800397 (2018). 10.1002/advs.201800397 10.1002/advs.201800397 PMC6096987 30128246

[95] R. Zhang, J. Zhao, G. Han, Z. Liu, C. Liu et al., Real-time discrimination and versatile profiling of spontaneous reactive oxygen species in living organisms with a single fluorescent probe. J. Am. Chem. Soc. **138**(11), 3769–3778 (2016). 10.1021/jacs.5b12848 26938117 10.1021/jacs.5b12848

[96] C. Zhao, Z. Li, J. Chen, L. Su, J. Wang et al., Site-specific biomimicry of antioxidative melanin formation and its application for acute liver injury therapy and imaging. Adv. Mater. **33**(34), e2102391 (2021). 10.1002/adma.202102391 34278624 10.1002/adma.202102391

[97] C. Zhao, J. Chen, J. Ye, Z. Li, L. Su et al., Structural transformative antioxidants for dual-responsive anti-inflammatory delivery and photoacoustic inflammation imaging. Angew. Chem. Int. Ed. **60**(26), 14458–14466 (2021). 10.1002/anie.202100873 10.1002/anie.202100873 33835672

[98] X. Ai, Z. Wang, H. Cheong, Y. Wang, R. Zhang et al., Multispectral optoacoustic imaging of dynamic redox correlation and pathophysiological progression utilizing upconversion nanoprobes. Nat. Commun. **10**(1), 1087 (2019). 10.1038/s41467-019-09001-7 30842426 10.1038/s41467-019-09001-7 PMC6403272

[99] Z. Lei, F. Zhang, Molecular engineering of NIR-II fluorophores for improved biomedical detection. Angew. Chem. Int. Ed. **60**(30), 16294–16308 (2021). 10.1002/anie.202007040 10.1002/anie.202007040 32780466

[100] C. Chu, J. Yu, E. Ren, S. Ou, Y. Zhang et al., Multimodal photoacoustic imaging-guided regression of corneal neovascularization: a non-invasive and safe strategy. Adv. Sci. **7**(14), 2000346 (2020). 10.1002/advs.202000346 10.1002/advs.202000346 PMC7375239 32714751

[101] Y. Ni, H. Liu, D. Dai, X. Mu, J. Xu et al., Chromogenic, fluorescent, and redox sensors for multichannel imaging and detection of hydrogen peroxide in living cell systems. Anal. Chem. **90**(17), 10152–10158 (2018). 10.1021/acs.analchem.7b04435 30058328 10.1021/acs.analchem.7b04435

[102] R. Singh, W. Zhang, X. Liu, B. Zhang, S. Kumar, Waveflex biosensor: MXene-Immobilized W-shaped fiber-based LSPR sensor for highly selective tyramine detection. Opt. Laser technol. **171**, 110357 (2024). 10.1016/j.optlastec.2023.110357

[103] H.C. Gomes, X. Liu, A. Fernandes, C. Moreirinha, R. Singh et al., Laser-induced graphene-based fabry-pérot cavity label-free immunosensors for the quantification of cortisol. Sens. Actuators Rep. **7**, 100186 (2024). 10.1016/j.snr.2024.100186

[104] R. Singh, W. Zhang, X. Liu, B. Zhang, S. Kumar, Humanoid-shaped waveflex biosensor for the detection of food contamination. Biomed. Opt. Express **14**(9), 4660–4676 (2023). 10.1364/boe.500311 37791266 10.1364/BOE.500311 PMC10545203

[105] G. Zhang, R. Singh, B. Zhang, S. Kumar, G. Li, Waveflex biosensor based on S-tapered and waist-expanded technique for detection of glycosylated hemoglobin. Biomed. Opt. Express **14**(11), 6100–6113 (2023). 10.1364/boe.505864 38021109 10.1364/BOE.505864 PMC10659782

[106] G. Li, X. Li, R. Singh, G. Zhang, B. Zhang et al., Slide-type waveflex biosensor based on signal enhancement technology for alpha-fetoprotein detection. Opt. Lett. **48**(18), 4745–4748 (2023). 10.1364/OL.501864 37707892 10.1364/OL.501864

[107] R. Singh, S. Kumar, S. Bera, S.K. Bhunia, Trends in using fluorescent mxene quantum dots for selective detection and bioimaging applications: a review. ACS Appl. Nano Mater. **6**(21), 19526–19550 (2023). 10.1021/acsanm.3c04491

[108] R. Singh, Z. Wang, C. Marques, R. Min, B. Zhang et al., Alanine aminotransferase detection using TIT assisted four tapered fiber structure-based LSPR sensor: from healthcare to marine life. Biosens. Bioelectron. **236**, 115424 (2023). 10.1016/j.bios.2023.115424 37253306 10.1016/j.bios.2023.115424

[109] A.M. Cameron, A. Castoldi, D.E. Sanin, L.J. Flachsmann, C.S. Field et al., Inflammatory macrophage dependence on NAD(+) salvage is a consequence of reactive oxygen species-mediated DNA damage. Nat. Immunol. **20**(4), 420–432 (2019). 10.1038/s41590-019-0336-y 30858618 10.1038/s41590-019-0336-y PMC12842115

[110] R.M. Palmer, A.G. Ferrige, S. Moncada, Nitric oxide release accounts for the biological activity of endothelium-derived relaxing factor. Nature **327**(6122), 524–526 (1987). 10.1038/327524a0 3495737 10.1038/327524a0

[111] E. Sasaki, H. Kojima, H. Nishimatsu, Y. Urano, K. Kikuchi et al., Highly sensitive near-infrared fluorescent probes for nitric oxide and their application to isolated organs. J. Am. Chem. Soc. **127**(11), 3684–3685 (2005). 10.1021/ja042967z 15771488 10.1021/ja042967z

[112] Y. Gabe, Y. Urano, K. Kikuchi, H. Kojima, T. Nagano, Highly sensitive fluorescence probes for nitric oxide based on boron dipyrromethene chromophore-rational design of potentially useful bioimaging fluorescence probe. J. Am. Chem. Soc. **126**(10), 3357–3367 (2004). 10.1021/ja037944j 15012166 10.1021/ja037944j

[113] H. Kojima, Y. Urano, K. Kikuchi, T. Higuchi, Y. Hirata et al., Fluorescent indicators for imaging nitric oxide production. Angew. Chem. Int. Ed. **38**(21), 3209–3212 (1999). 10.1002/(sici)1521-3773(19991102)38:21%3c3209::aid-anie3209%3e3.0.co;2-6 10.1002/(sici)1521-3773(19991102)38:21<3209::aid-anie3209>3.0.co;2-6 10556905

[114] M.M. Sadek, M.B.A. Olia, C.J. Nowell, N. Barlow, C.H. Schiesser et al., Characterisation of a novel coumarin-based fluorescent probe for monitoring nitric oxide production in macrophages. Bioorgan. Med. Chem. **25**(20), 5743–5748 (2017). 10.1016/j.bmc.2017.08.054 10.1016/j.bmc.2017.08.054 28927902

[115] E.Y. Zhou, H.J. Knox, C.J. Reinhardt, G. Partipilo, M.J. Nilges et al., Near-infrared photoactivatable nitric oxide donors with integrated photoacoustic monitoring. J. Am. Chem. Soc. **140**(37), 11686–11697 (2018). 10.1021/jacs.8b05514 30198716 10.1021/jacs.8b05514 PMC7331458

[116] Z. Mao, H. Jiang, Z. Li, C. Zhong, W. Zhang et al., An N-nitrosation reactivity-based two-photon fluorescent probe for the specific in situ detection of nitric oxide. Chem. Sci. **8**(6), 4533–4538 (2017). 10.1039/c7sc00416h 28660066 10.1039/c7sc00416h PMC5472031

[117] J. Miao, Y. Huo, Q. Liu, Z. Li, H. Shi et al., A new class of fast-response and highly selective fluorescent probes for visualizing peroxynitrite in live cells, subcellular organelles, and kidney tissue of diabetic rats. Biomaterials **107**, 33–43 (2016). 10.1016/j.biomaterials.2016.08.032 27606902 10.1016/j.biomaterials.2016.08.032

[118] Y. Huo, J. Miao, Y. Li, Y. Shi, H. Shi et al., Aromatic primary monoamine-based fast-response and highly specific fluorescent probes for imaging the biological signaling molecule nitric oxide in living cells and organisms. J. Mater. Chem. B **5**(13), 2483–2490 (2017). 10.1039/c6tb03382b 32264554 10.1039/c6tb03382b

[119] T.W. Shiue, Y.H. Chen, C.M. Wu, G. Singh, H.Y. Chen et al., Nitric oxide turn-on fluorescent probe based on deamination of aromatic primary monoamines. Inorg. Chem. **51**(9), 5400–5408 (2012). 10.1021/ic300379u 22486484 10.1021/ic300379u

[120] A. Fernandez, M. Vendrell, Smart fluorescent probes for imaging macrophage activity. Chem. Soc. Rev. **45**(5), 1182–1196 (2016). 10.1039/c5cs00567a 26752349 10.1039/c5cs00567a

[121] P. Yuan, X. Xu, D. Hu, Y. Chen, J. Fan et al., Highly sensitive imaging of tumor metastasis based on the targeting and polarization of M2-like macrophages. J. Am. Chem. Soc. **145**(14), 7941–7951 (2023). 10.1021/jacs.2c13218 36987634 10.1021/jacs.2c13218

[122] Y. Huo, J. Miao, J. Fang, H. Shi, J. Wang et al., Aromatic secondary amine-functionalized fluorescent NO probes: improved detection sensitivity for NO and potential applications in cancer immunotherapy studies. Chem. Sci. **10**(1), 145–152 (2019). 10.1039/c8sc03694b 30713625 10.1039/c8sc03694b PMC6328002

[123] A. Ramesh, S. Kumar, A. Brouillard, D. Nandi, A. Kulkarni, A nitric oxide (NO) nanoreporter for noninvasive real-time imaging of macrophage immunotherapy. Adv. Mater. **32**(24), e2000648 (2020). 10.1002/adma.202000648 32390270 10.1002/adma.202000648

[124] R. Radi, Peroxynitrite, a stealthy biological oxidant. J. Biol. Chem. **288**(37), 26464–26472 (2013). 10.1074/jbc.R113.472936 23861390 10.1074/jbc.R113.472936 PMC3772193

[125] J. Kim, J. Park, H. Lee, Y. Choi, Y. Kim, A boronate-based fluorescent probe for the selective detection of cellular peroxynitrite. Chem. Commun. **50**(66), 9353–9356 (2014). 10.1039/c4cc02943g 10.1039/c4cc02943g 25002151

[126] J. Peng, A. Samanta, X. Zeng, S. Han, L. Wang et al., Real-time in vivo hepatotoxicity monitoring through chromophore-conjugated photon-upconverting nanoprobes. Angew. Chem. Int. Ed. **56**(15), 4165–4169 (2017). 10.1002/anie.201612020 10.1002/anie.201612020 28295935

[127] C. Wang, W. Fan, Z. Zhang, Y. Wen, L. Xiong et al., Advanced nanotechnology leading the way to multimodal imaging-guided precision surgical therapy. Adv. Mater. **31**(49), e1904329 (2019). 10.1002/adma.201904329 31538379 10.1002/adma.201904329

[128] L. Wu, Y. Ishigaki, W. Zeng, T. Harimoto, B. Yin et al., Generation of hydroxyl radical-activatable ratiometric near-infrared bimodal probes for early monitoring of tumor response to therapy. Nat. Commun. **12**(1), 6145 (2021). 10.1038/s41467-021-26380-y 34686685 10.1038/s41467-021-26380-y PMC8536768

[129] Y. Wang, Y. Zhang, Z. Wang, J. Zhang, R.R. Qiao et al., Optical/MRI dual-modality imaging of M1 macrophage polarization in atherosclerotic plaque with marco-targeted upconversion luminescence probe. Biomaterials **219**, 119378 (2019). 10.1016/j.biomaterials.2019.119378 31382209 10.1016/j.biomaterials.2019.119378

[130] L. Yuan, W. Lin, Y. Yang, H. Chen, A unique class of near-infrared functional fluorescent dyes with carboxylic-acid-modulated fluorescence ON/OFF switching: rational design, synthesis, optical properties, theoretical calculations, and applications for fluorescence imaging in living animals. J. Am. Chem. Soc. **134**(2), 1200–1211 (2012). 10.1021/ja209292b 22176300 10.1021/ja209292b

[131] M. Schieber, N.S. Chandel, ROS function in redox signaling and oxidative stress. Curr. Biol. **24**(10), R453–R462 (2014). 10.1016/j.cub.2014.03.034 24845678 10.1016/j.cub.2014.03.034 PMC4055301

[132] G. Morris, M. Gevezova, V. Sarafian, M. Maes, Redox regulation of the immune response. Cell. Mol. Immunol. **19**(10), 1079–1101 (2022). 10.1038/s41423-022-00902-0 36056148 10.1038/s41423-022-00902-0 PMC9508259

[133] J.S. Arthur, S.C. Ley, Mitogen-activated protein kinases in innate immunity. Nat. Rev. Immunol. **13**(9), 679–692 (2013). 10.1038/nri3495 23954936 10.1038/nri3495

[134] H. Kamata, S. Honda, S. Maeda, L.F. Chang, H. Hirata et al., Reactive oxygen species promote TNFα-induced death and sustained JNK activation by inhibiting MAP kinase phosphatases. Cell **120**(5), 649–661 (2005). 10.1016/j.cell.2004.12.041 15766528 10.1016/j.cell.2004.12.041

[135] C. Wu, W. Zhao, X. Zhang, X. Chen, Neocryptotanshinone inhibits lipopolysaccharide-induced inflammation in RAW 264.7 macrophages by suppression of NF-kappaB and iNOS signaling pathways. Acta. Pharm. Sin. B **5**(4), 323–329 (2015). 10.1016/j.apsb.2015.01.010 26579462 10.1016/j.apsb.2015.01.010 PMC4629269

[136] C. Bogdan, Nitric oxide and the immune response. Nat. Immunol. **2**(10), 907–916 (2001). 10.1038/ni1001-907 11577346 10.1038/ni1001-907

[137] V. Toshchakov, B.W. Jones, P.Y. Perera, K. Thomas, M.J. Cody et al., TLR4, but not TLR2, mediates IFN-beta-induced STAT1alpha/beta-dependent gene expression in macrophages. Nat. Immunol. **3**(4), 392–398 (2002). 10.1038/ni774 11896392 10.1038/ni774

[138] J.S. Ma, W.J. Kim, J.J. Kim, T.J. Kim, S.K. Ye et al., Gold nanoparticles attenuate LPS-induced NO production through the inhibition of NF-kappab and IFN-beta/STAT1 pathways in RAW264.7 cells. Nitric Oxide **23**(3), 214–219 (2010). 10.1016/j.niox.2010.06.005 20547236 10.1016/j.niox.2010.06.005

[139] E. Butturini, D. Boriero, A. Carcereri de Prati, S. Mariotto, STAT1 drives M1 microglia activation and neuroinflammation under hypoxia. Arch. Biochem. Biophys. **669**, 22–30 (2019). 10.1016/j.abb.2019.05.011 31121156 10.1016/j.abb.2019.05.011

[140] B. Everts, E. Amiel, G.J. van der Windt, T.C. Freitas, R. Chott et al., Commitment to glycolysis sustains survival of NO-producing inflammatory dendritic cells. Blood **120**(7), 1422–1431 (2012). 10.1182/blood-2012-03-419747 22786879 10.1182/blood-2012-03-419747 PMC3423780

[141] Q. Zhang, D. Dehaini, Y. Zhang, J. Zhou, X. Chen et al., Neutrophil membrane-coated nanoparticles inhibit synovial inflammation and alleviate joint damage in inflammatory arthritis. Nat. Nanotechnol. **13**(12), 1182–1190 (2018). 10.1038/s41565-018-0254-4 30177807 10.1038/s41565-018-0254-4

[142] W. Yang, Y. Tao, Y. Wu, X. Zhao, W. Ye et al., Neutrophils promote the development of reparative macrophages mediated by ROS to orchestrate liver repair. Nat. Commun. **10**(1), 1076 (2019). 10.1038/s41467-019-09046-8 30842418 10.1038/s41467-019-09046-8 PMC6403250

[143] A. Gombozhapova, Y. Rogovskaya, V. Shurupov, M. Rebenkova, J. Kzhyshkowska et al., Macrophage activation and polarization in post-infarction cardiac remodeling. J. Biomed. Sci. **24**(1), 13 (2017). 10.1186/s12929-017-0322-3 28173864 10.1186/s12929-017-0322-3 PMC5297120

[144] E.H. Choo, J.H. Lee, E.H. Park, H.E. Park, N.C. Jung et al., Infarcted myocardium-primed dendritic cells improve remodeling and cardiac function after myocardial infarction by modulating the regulatory T cell and macrophage polarization. Circulation **135**(15), 1444–1457 (2017). 10.1161/CIRCULATIONAHA.116.023106 28174192 10.1161/CIRCULATIONAHA.116.023106

[145] Y. Liu, K. Ai, X. Ji, D. Askhatova, R. Du et al., Comprehensive insights into the multi-antioxidative mechanisms of melanin nanoparticles and their application to protect brain from injury in ischemic stroke. J. Am. Chem. Soc. **139**(2), 856–862 (2017). 10.1021/jacs.6b11013 27997170 10.1021/jacs.6b11013 PMC5752099

[146] X. Bao, J. Zhao, J. Sun, M. Hu, X. Yang, Polydopamine nanoparticles as efficient scavengers for reactive oxygen species in periodontal disease. ACS Nano **12**(9), 8882–8892 (2018). 10.1021/acsnano.8b04022 30028940 10.1021/acsnano.8b04022

[147] C. Zhang, X. Wang, J. Du, Z. Gu, Y. Zhao, Reactive oxygen species-regulating strategies based on nanomaterials for disease treatment. Adv. Sci. **8**(3), 2002797 (2021). 10.1002/advs.202002797 10.1002/advs.202002797 PMC7856897 33552863

[148] L. Zhang, Q.C. Yang, S. Wang, Y. Xiao, S.C. Wan et al., Engineering multienzyme-mimicking covalent organic frameworks as pyroptosis inducers for boosting antitumor immunity. Adv. Mater. **34**(13), e2108174 (2022). 10.1002/adma.202108174 34918837 10.1002/adma.202108174

[149] J. Zhang, B. Gao, B. Ye, Z. Sun, Z. Qian et al., Mitochondrial-targeted delivery of polyphenol-mediated antioxidases complexes against pyroptosis and inflammatory diseases. Adv. Mater. **35**(11), e2208571 (2023). 10.1002/adma.202208571 36648306 10.1002/adma.202208571

[150] T. Liu, B. Xiao, F. Xiang, J. Tan, Z. Chen et al., Ultrasmall copper-based nanoparticles for reactive oxygen species scavenging and alleviation of inflammation related diseases. Nat. Commun. **11**(1), 2788 (2020). 10.1038/s41467-020-16544-7 32493916 10.1038/s41467-020-16544-7 PMC7270130

[151] Y. Peng, D. He, X. Ge, Y. Lu, Y. Chai et al., Construction of heparin-based hydrogel incorporated with Cu_5.4_O ultrasmall nanozymes for wound healing and inflammation inhibition. Bioact. Mater. **6**(10), 3109–3124 (2021). 10.1016/j.bioactmat.2021.02.006 33778192 10.1016/j.bioactmat.2021.02.006 PMC7960791

[152] M. Soh, D.W. Kang, H.G. Jeong, D. Kim, D.Y. Kim et al., Ceria-Zirconia nanoparticles as an enhanced multi-antioxidant for sepsis treatment. Angew. Chem. Int. Ed. **56**(38), 11399–11403 (2017). 10.1002/anie.201704904 10.1002/anie.201704904 28643857

[153] S.I. Han, S.W. Lee, M.G. Cho, J.M. Yoo, M.H. Oh et al., Epitaxially strained CeO_2_/Mn_3_O_4_ nanocrystals as an enhanced antioxidant for radioprotection. Adv. Mater. **32**(31), e2001566 (2020). 10.1002/adma.202001566 32520432 10.1002/adma.202001566

[154] X. Zhou, Q. Zhou, Z. He, Y. Xiao, Y. Liu et al., ROS balance autoregulating core-shell CeO_2_@ZIF-8/Au nanoplatform for wound repair. Nano-Micro Lett. **16**(1), 156 (2024). 10.1007/s40820-024-01353-0 10.1007/s40820-024-01353-0 PMC10957853 38512388

[155] J. Kim, H.Y. Kim, S.Y. Song, S.H. Go, H.S. Sohn et al., Synergistic oxygen generation and reactive oxygen species scavenging by manganese ferrite/ceria co-decorated nanoparticles for rheumatoid arthritis treatment. ACS Nano **13**(3), 3206–3217 (2019). 10.1021/acsnano.8b08785 30830763 10.1021/acsnano.8b08785

[156] J. Mu, C. Li, Y. Shi, G. Liu, J. Zou et al., Protective effect of platinum nano-antioxidant and nitric oxide against hepatic ischemia-reperfusion injury. Nat. Commun. **13**(1), 2513 (2022). 10.1038/s41467-022-29772-w 35523769 10.1038/s41467-022-29772-w PMC9076604

[157] X.D. Zhang, X.K. Chen, Y.L. Zhao, Nanozymes: versatile platforms for cancer diagnosis and therapy. Nano-Micro Lett. **14**(1), 95 (2022). 10.1007/s40820-022-00828-2 10.1007/s40820-022-00828-2 PMC8986955 35384520

[158] Y.J. Li, S.J.Y. Gao, S.R. Shi, D.X. Xiao, S.L. Peng et al., Tetrahedral framework nucleic acid-based delivery of resveratrol alleviates insulin resistance: from innate to adaptive immunity. Nano-Micro Lett. **13**(1), 186 (2021). 10.1007/s40820-021-00711-6 10.1007/s40820-021-00614-6 PMC8006527 34138319

[159] F. Zhou, M. Li, M. Chen, M. Chen, X. Chen et al., Redox homeostasis strategy for inflammatory macrophage reprogramming in rheumatoid arthritis based on ceria oxide nanozyme-complexed biopolymeric micelles. ACS Nano **17**(5), 4358–4372 (2023). 10.1021/acsnano.2c09127 36847819 10.1021/acsnano.2c09127

[160] Y. Guo, Q. Sun, F.G. Wu, Y. Dai, X. Chen, Polyphenol-containing nanoparticles: synthesis, properties, and therapeutic delivery. Adv. Mater. **33**(22), e2007356 (2021). 10.1002/adma.202007356 33876449 10.1002/adma.202007356

[161] M.S. Petronek, J.M. Stolwijk, S.D. Murray, E.J. Steinbach, Y. Zakharia et al., Utilization of redox modulating small molecules that selectively act as pro-oxidants in cancer cells to open a therapeutic window for improving cancer therapy. Redox Biol. **42**, 101864 (2021). 10.1016/j.redox.2021.101864 33485837 10.1016/j.redox.2021.101864 PMC8113052

[162] W.L. Wan, Y.J. Lin, H.L. Chen, C.C. Huang, P.C. Shih et al., In situ nanoreactor for photosynthesizing H_2_ gas to mitigate oxidative stress in tissue inflammation. J. Am. Chem. Soc. **139**(37), 12923–12926 (2017). 10.1021/jacs.7b07492 28870078 10.1021/jacs.7b07492

[163] B. Zhao, L. Zeng, D. Chen, S. Xie, Z. Jin et al., NIR-photocatalytic regulation of arthritic synovial microenvironment. Sci. Adv. **8**(40), eabq0959 (2022). 10.1126/sciadv.abq0959 36197972 10.1126/sciadv.abq0959 PMC9534508

[164] G. Yang, J.S. Ni, Y. Li, M. Zha, Y. Tu et al., Acceptor engineering for optimized ROS generation facilitates reprogramming macrophages to M1 phenotype in photodynamic immunotherapy. Angew. Chem. Int. Ed. **60**(10), 5386–5393 (2021). 10.1002/anie.202013228 10.1002/anie.202013228 33236483

[165] M.W. Cleeter, J.M. Cooper, V.M. Darley-Usmar, S. Moncada, A.H. Schapira, Reversible inhibition of cytochrome c oxidase, the terminal enzyme of the mitochondrial respiratory chain, by nitric oxide. Implications for neurodegenerative diseases. FEBS Lett. **345**(1), 50–54 (1994). 10.1016/0014-5793(94)00424-2 8194600 10.1016/0014-5793(94)00424-2

[166] S. Theivendran, Z. Gu, J. Tang, Y. Yang, H. Song et al., Nanostructured organosilica nitric oxide donors intrinsically regulate macrophage polarization with antitumor effect. ACS Nano **16**(7), 10943–10957 (2022). 10.1021/acsnano.2c03348 35735363 10.1021/acsnano.2c03348

[167] M. Liang, X. Yan, Nanozymes: from new concepts, mechanisms, and standards to applications. Acc. Chem. Res. **52**(8), 2190–2200 (2019). 10.1021/acs.accounts.9b00140 31276379 10.1021/acs.accounts.9b00140

[168] K. Sridhar, B.S. Inbaraj, B.H. Chen, Recent advances on nanoparticle based strategies for improving carotenoid stability and biological activity. Antioxidants **10**(5), 713 (2021). 10.3390/antiox10050713 33946470 10.3390/antiox10050713 PMC8147144

[169] D. Zhong, Z. Du, M. Zhou, Algae: a natural active material for biomedical applications. View **2**(4), 20200189 (2021). 10.1002/viw.20200189

[170] H. Chen, Y. Guo, Z. Zhang, W. Mao, C. Shen et al., Symbiotic algae-bacteria dressing for producing hydrogen to accelerate diabetic wound healing. Nano Lett. **22**(1), 229–237 (2022). 10.1021/acs.nanolett.1c03693 34928162 10.1021/acs.nanolett.1c03693

[171] W. Geng, X. Liu, B. Tao, Y. He, K. Li et al., Nitric oxide scavenging and hydrogen sulfide production synergistically treat rheumatoid arthritis. Adv. Healthc. Mater. **12**(4), 2202380 (2023). 10.1002/adhm.202202380 10.1002/adhm.202202380 36337007

[172] Y. Yan, A. Lu, Y. Dou, Z. Zhang, X.Y. Wang et al., Nanomedicines reprogram synovial macrophages by scavenging nitric oxide and silencing CA9 in progressive osteoarthritis. Adv. Sci. **10**(11), 2207490 (2023). 10.1002/advs.202207490 10.1002/advs.202207490 PMC10104675 36748885

[173] F. Zhou, J. Mei, S. Yang, X. Han, H. Li et al., Modified ZIF-8 nanoparticles attenuate osteoarthritis by reprogramming the metabolic pathway of synovial macrophages. ACS Appl. Mater. Interfaces **12**(2), 2009–2022 (2020). 10.1021/acsami.9b16327 31849213 10.1021/acsami.9b16327

[174] M. He, L. Sun, X. Fu, S.P. McDonough, C.C. Chu, Biodegradable amino acid-based poly(ester amine) with tunable immunomodulating properties and their in vitro and in vivo wound healing studies in diabetic rats’ wounds. Acta Biomater. **84**, 114–132 (2019). 10.1016/j.actbio.2018.11.053 30508656 10.1016/j.actbio.2018.11.053

[175] X. Li, X. Wang, Q. Liu, J. Yan, D. Pan et al., ROS-responsive boronate-stabilized polyphenol-poloxamer 188 assembled dexamethasone nanodrug for macrophage repolarization in osteoarthritis treatment. Adv. Healthc. Mater. **10**(20), 2100883 (2021). 10.1002/adhm.202100883 10.1002/adhm.202100883 34137218

[176] L. Valls-Lacalle, I. Barba, E. Miro-Casas, J.J. Alburquerque-Bejar, M. Ruiz-Meana et al., Succinate dehydrogenase inhibition with malonate during reperfusion reduces infarct size by preventing mitochondrial permeability transition. Cardiovasc. Res. **109**(3), 374–384 (2016). 10.1093/cvr/cvv279 26705364 10.1093/cvr/cvv279

[177] L. Davenport Huyer, S. Mandla, Y. Wang, S.B. Campbell, B. Yee et al., Macrophage immunomodulation through new polymers that recapitulate functional effects of itaconate as a power house of innate immunity. Adv. Funct. Mater. **31**(6), 2003341 (2020). 10.1002/adfm.202003341 33708036 10.1002/adfm.202003341 PMC7942808

[178] J.R. Nakkala, Y. Yao, Z. Zhai, Y. Duan, D. Zhang et al., Dimethyl itaconate-loaded nanofibers rewrite macrophage polarization, reduce inflammation, and enhance repair of myocardic infarction. Small **17**(17), 2006992 (2021). 10.1002/smll.202006992 10.1002/smll.202006992 33719217

[179] F. Zhu, L. Zi, P. Yang, Y. Wei, R. Zhong et al., Efficient iron and ROS nanoscavengers for brain protection after intracerebral hemorrhage. ACS Appl. Mater. Interfaces **13**(8), 9729–9738 (2021). 10.1021/acsami.1c00491 33599495 10.1021/acsami.1c00491

[180] X. Dong, P. Wu, L. Yan, K. Liu, W. Wei et al., Oriented nanofibrous P(MMD-co-LA)/deferoxamine nerve scaffold facilitates peripheral nerve regeneration by regulating macrophage phenotype and revascularization. Biomaterials **280**, 121288 (2022). 10.1016/j.biomaterials.2021.121288 34894585 10.1016/j.biomaterials.2021.121288

[181] J. Han, Y.S. Kim, M.Y. Lim, H.Y. Kim, S. Kong et al., Dual roles of graphene oxide to attenuate inflammation and elicit timely polarization of macrophage phenotypes for cardiac repair. ACS Nano **12**(2), 1959–1977 (2018). 10.1021/acsnano.7b09107 29397689 10.1021/acsnano.7b09107

[182] Y. Li, X. Teng, C. Yang, Y. Wang, L. Wang et al., Ultrasound controlled anti-inflammatory polarization of platelet decorated microglia for targeted ischemic stroke therapy. Angew. Chem. Int. Ed. **60**(10), 5083–5090 (2021). 10.1002/anie.202010391 10.1002/anie.202010391 33259112

[183] L. Chen, L. Zhang, H. Zhang, X. Sun, D. Liu et al., Programmable immune activating electrospun fibers for skin regeneration. Bioactive. Mater. **6**(10), 3218–3230 (2021). 10.1016/j.bioactmat.2021.02.022 10.1016/j.bioactmat.2021.02.022 PMC7966852 33778200

[184] T.T. Tang, B. Wang, M. Wu, Z.L. Li, Y. Feng et al., Extracellular vesicle-encapsulated IL-10 as novel nanotherapeutics against ischemic AKI. Sci. Adv. **6**(33), eaaz0748 (2020). 10.1126/sciadv.aaz0748 32851154 10.1126/sciadv.aaz0748 PMC7423360

[185] H. Kang, K. Zhang, D.S.H. Wong, F. Han, B. Li et al., Near-infrared light-controlled regulation of intracellular calcium to modulate macrophage polarization. Biomaterials **178**, 681–696 (2018). 10.1016/j.biomaterials.2018.03.007 29705000 10.1016/j.biomaterials.2018.03.007

[186] X. Lei, G. Tan, Y. Wang, L. Chen, Y. Cao et al., Mitochondrial calcium nanoregulators reverse the macrophage proinflammatory phenotype through restoring mitochondrial calcium homeostasis for the treatment of osteoarthritis. Int. J. Nanomedicine **18**, 1469–1489 (2023). 10.2147/IJN.S402170 36998601 10.2147/IJN.S402170 PMC10046163

[187] J. Wu, M. Wang, Y. Wu, J. Liu, W. Zhi et al., Coupling BCP ceramics with micro-vibration stimulation field for cascade amplification from immune activation to bone regeneration. Adv. Funct. Mater. **33**, 2215079 (2023). 10.1002/adfm.202215079

[188] N. Feng, L. Liang, M. Fan, Y. Du, C. Chen et al., Treating autoimmune inflammatory diseases with an siERN1-nanoprodrug that mediates macrophage polarization and blocks Toll-like receptor signaling. ACS Nano **15**(10), 15874–15891 (2021). 10.1021/acsnano.1c03726 34586802 10.1021/acsnano.1c03726

[189] N. Li, Q. Du, Z. Jing, L. Xue, W. He et al., Study of the effects of Au@ZIF-8 on metabolism in mouse RAW 264.7 macrophages. Biomater. Adv. **138**, 212800 (2022). 10.1016/j.bioadv.2022.212800 35913225 10.1016/j.bioadv.2022.212800

[190] H. Gu, Y. Zhu, J. Yang, R. Jiang, Y. Deng et al., Liver-inspired polyetherketoneketone scaffolds simulate regenerative signals and mobilize anti-inflammatory reserves to reprogram macrophage metabolism for boosted osteoporotic osseointegration. Adv. Sci. **10**(25), e2302136 (2023). 10.1002/advs.202302136 10.1002/advs.202302136 PMC10477864 37400369

[191] Y. Sang, Q. Deng, F. Cao, Z. Liu, Y. You et al., Remodeling macrophages by an iron nanotrap for tumor growth suppression. ACS Nano **15**(12), 19298–19309 (2021). 10.1021/acsnano.1c05392 34783526 10.1021/acsnano.1c05392

[192] Y. Yang, Q. Tian, S. Wu, Y. Li, K. Yang et al., Blue light-triggered Fe^2+^-release from monodispersed ferrihydrite nanoparticles for cancer iron therapy. Biomaterials **271**, 120739 (2021). 10.1016/j.biomaterials.2021.120739 33690102 10.1016/j.biomaterials.2021.120739

[193] C.X. Li, Y. Zhang, X. Dong, L. Zhang, M.D. Liu et al., Artificially reprogrammed macrophages as tumor-tropic immunosuppression-resistant biologics to realize therapeutics production and immune activation. Adv. Mater. **31**(15), e1807211 (2019). 10.1002/adma.201807211 30803083 10.1002/adma.201807211

[194] M.H. Oh, I.H. Sun, L. Zhao, R.D. Leone, I.M. Sun et al., Targeting glutamine metabolism enhances tumor-specific immunity by modulating suppressive myeloid cells. J. Clin. Invest. **130**(7), 3865–3884 (2020). 10.1172/jci131859 32324593 10.1172/JCI131859 PMC7324212

[195] X.B. Shulan Han, Y. Zou, L. Wang, Y. Li, L. Yang et al., D-lactate modulates M2 tumor-associated macrophages and remodels immunosuppressive tumor microenvironment for hepatocellular carcinoma. Sci. Adv. **9**(29), eadg2697 (2023). 10.1126/sciadv.adg2697 37467325 10.1126/sciadv.adg2697 PMC10355835

[196] J. Zhao, Z. Tian, S. Zhao, D. Feng, Z. Guo et al., Insights into the effect of catalytic intratumoral lactate depletion on metabolic reprogramming and immune activation for antitumoral activity. Adv. Sci. **10**(4), e2204808 (2023). 10.1002/advs.202204808 10.1002/advs.202204808 PMC9896070 36479819

[197] Z.Y. Han, C. Zhang, J.X. An, Y.Z. Wang, J.Y. Qiao et al., Metabolic regulation of tumor microenvironment with biohybrid bacterial bioreactor for enhanced cancer chemo-immunotherapy. Adv. Funct. Mater. **33**, 2302728 (2023). 10.1002/adfm.202302728

[198] G.M. Tannahill, A.M. Curtis, J. Adamik, E.M. Palsson-McDermott, A.F. McGettrick et al., Succinate is an inflammatory signal that induces IL-1β through HIF-1alpha. Nature **496**(7444), 238–242 (2013). 10.1038/nature11986 23535595 10.1038/nature11986 PMC4031686

[199] M.P. Murphy, L.A.J. O’Neill, Krebs cycle reimagined: The emerging roles of succinate and itaconate as signal transducers. Cell **174**(4), 780–784 (2018). 10.1016/j.cell.2018.07.030 30096309 10.1016/j.cell.2018.07.030

[200] E.T. Chouchani, V.R. Pell, E. Gaude, D. Aksentijevic, S.Y. Sundier et al., Ischaemic accumulation of succinate controls reperfusion injury through mitochondrial ROS. Nature **515**(7527), 431–435 (2014). 10.1038/nature13909 25383517 10.1038/nature13909 PMC4255242

[201] C.L. Strelko, W. Lu, F.J. Dufort, T.N. Seyfried, T.C. Chiles et al., Itaconic acid is a mammalian metabolite induced during macrophage activation. J. Am. Chem. Soc. **133**(41), 16386–16389 (2011). 10.1021/ja2070889 21919507 10.1021/ja2070889 PMC3216473

[202] A. Michelucci, T. Cordes, J. Ghelfi, A. Pailot, N. Reiling et al., Immune-responsive gene 1 protein links metabolism to immunity by catalyzing itaconic acid production. Proc. Natl. Acad. Sci. U.S.A. **110**(19), 7820–7825 (2013). 10.1073/pnas.1218599110 23610393 10.1073/pnas.1218599110 PMC3651434

[203] W. Qin, K. Qin, Y. Zhang, W. Jia, Y. Chen et al., S-glycosylation-based cysteine profiling reveals regulation of glycolysis by itaconate. Nat. Chem. Biol. **15**(10), 983–991 (2019). 10.1038/s41589-019-0323-5 31332308 10.1038/s41589-019-0323-5

[204] W. Qin, Y. Zhang, H. Tang, D. Liu, Y. Chen et al., Chemoproteomic profiling of itaconation by bioorthogonal probes in inflammatory macrophages. J. Am. Chem. Soc. **142**(25), 10894–10898 (2020). 10.1021/jacs.9b11962 32496768 10.1021/jacs.9b11962

[205] P. Sun, Z. Zhang, B. Wang, C. Liu, C. Chen et al., A genetically encoded fluorescent biosensor for detecting itaconate with subcellular resolution in living macrophages. Nat. Commun. **13**(1), 6562 (2022). 10.1038/s41467-022-34306-5 36333306 10.1038/s41467-022-34306-5 PMC9636186

[206] A. Hooftman, L.A.J. O’Neill, The immunomodulatory potential of the metabolite itaconate. Trends Immunol. **40**(8), 687–698 (2019). 10.1016/j.it.2019.05.007 31178405 10.1016/j.it.2019.05.007

[207] V. Lampropoulou, A. Sergushichev, M. Bambouskova, S. Nair, E.E. Vincent et al., Itaconate links inhibition of succinate dehydrogenase with macrophage metabolic remodeling and regulation of inflammation. Cell Metab. **24**(1), 158–166 (2016). 10.1016/j.cmet.2016.06.004 27374498 10.1016/j.cmet.2016.06.004 PMC5108454

[208] L.A.J. O’Neill, M.N. Artyomov, Itaconate: the poster child of metabolic reprogramming in macrophage function. Nat. Rev. Immunol. **19**(5), 273–281 (2019). 10.1038/s41577-019-0128-5 30705422 10.1038/s41577-019-0128-5

[209] E.L. Mills, D.G. Ryan, H.A. Prag, D. Dikovskaya, D. Menon et al., Itaconate is an anti-inflammatory metabolite that activates Nrf2 via alkylation of KEAP1. Nature **556**(7699), 113–117 (2018). 10.1038/nature25986 29590092 10.1038/nature25986 PMC6047741

[210] M. Bambouskova, L. Gorvel, V. Lampropoulou, A. Sergushichev, E. Loginicheva et al., Electrophilic properties of itaconate and derivatives regulate the Ikappabzeta-ATF3 inflammatory axis. Nature **556**(7702), 501–504 (2018). 10.1038/s41586-018-0052-z 29670287 10.1038/s41586-018-0052-z PMC6037913

[211] L. Ni, Z. Lin, S. Hu, Y. Shi, Z. Jiang et al., Itaconate attenuates osteoarthritis by inhibiting STING/NF-kappaB axis in chondrocytes and promoting M2 polarization in macrophages. Biochem. Pharmacol. **198**, 114935 (2022). 10.1016/j.bcp.2022.114935 35104478 10.1016/j.bcp.2022.114935

[212] H. Yu, P. Ren, X. Pan, X. Zhang, J. Ma et al., Intracellular delivery of itaconate by metal-organic framework-anchored hydrogel microspheres for osteoarthritis therapy. Pharmaceutics **15**(3), 724 (2023). 10.3390/pharmaceutics15030724 36986584 10.3390/pharmaceutics15030724 PMC10051475

[213] Q. Ding, T. Sun, W. Su, X. Jing, B. Ye et al., Bioinspired multifunctional black phosphorus hydrogel with antibacterial and antioxidant properties: a stepwise countermeasure for diabetic skin wound healing. Adv. Healthc. Mater. **11**(12), e2102791 (2022). 10.1002/adhm.202102791 35182097 10.1002/adhm.202102791

[214] V.L. Nelson, H.C.B. Nguyen, J.C. Garcia-Canaveras, E.R. Briggs, W.Y. Ho et al., PPARgamma is a nexus controlling alternative activation of macrophages via glutamine metabolism. Genes Dev. **32**(15–16), 1035–1044 (2018). 10.1101/gad.312355.118 30006480 10.1101/gad.312355.118 PMC6075146

[215] M.C. Runtsch, S. Angiari, A. Hooftman, R. Wadhwa, Y. Zhang et al., Itaconate and itaconate derivatives target JAK1 to suppress alternative activation of macrophages. Cell Metab. **34**(3), 487–50.e18 (2022). 10.1016/j.cmet.2022.02.002 35235776 10.1016/j.cmet.2022.02.002

[216] V.C. Ganta, M.H. Choi, A. Kutateladze, T.E. Fox, C.R. Farber et al., A microRNA93-interferon regulatory factor-9-immunoresponsive gene-1-itaconic acid pathway modulates M2-like macrophage polarization to revascularize ischemic muscle. Circulation **135**(24), 2403–2425 (2017). 10.1161/CIRCULATIONAHA.116.025490 28356443 10.1161/CIRCULATIONAHA.116.025490 PMC5503157

[217] T. Ganz, E. Nemeth, Iron homeostasis in host defence and inflammation. Nat. Rev. Immunol. **15**(8), 500–510 (2015). 10.1038/nri3863 26160612 10.1038/nri3863 PMC4801113

[218] S. Recalcati, M. Locati, A. Marini, P. Santambrogio, F. Zaninotto et al., Differential regulation of iron homeostasis during human macrophage polarized activation. Eur. J. Immunol. **40**(3), 824–835 (2010). 10.1002/eji.200939889 20039303 10.1002/eji.200939889

[219] C. Pfeifhofer-Obermair, P. Tymoszuk, V. Petzer, G. Weiss, M. Nairz, Iron in the tumor microenvironment-connecting the dots. Front. Oncol. **8**, 549 (2018). 10.3389/fonc.2018.00549 30534534 10.3389/fonc.2018.00549 PMC6275298

[220] M. Jung, C. Mertens, B. Brune, Macrophage iron homeostasis and polarization in the context of cancer. Immunobiology **220**(2), 295–304 (2015). 10.1016/j.imbio.2014.09.011 25260218 10.1016/j.imbio.2014.09.011

[221] S. Recalcati, M. Locati, E. Gammella, P. Invernizzi, G. Cairo, Iron levels in polarized macrophages: regulation of immunity and autoimmunity. Autoimmun. Rev. **11**(12), 883–889 (2012). 10.1016/j.autrev.2012.03.003 22449938 10.1016/j.autrev.2012.03.003

[222] A.T. Aron, A.G. Reeves, C.J. Chang, Activity-based sensing fluorescent probes for iron in biological systems. Curr. Opin. Chem. Biol. **43**, 113–118 (2018). 10.1016/j.cbpa.2017.12.010 29306820 10.1016/j.cbpa.2017.12.010 PMC5847483

[223] W. Xing, H. Xu, H. Ma, S.A.A. Abedi, S. Wang et al., A PET-based fluorescent probe for monitoring labile Fe(II) pools in macrophage activations and ferroptosis. Chem. Commun. **58**(18), 2979–2982 (2022). 10.1039/d1cc06611k 10.1039/d1cc06611k 35147150

[224] H.Y. Lin, R. Weissleder, E. Corradini, J.L. Babitt, Y. Xia et al., Method for measuring macrophage iron efflux in vitro and in vivo using magnetic resonance imaging. Blood **112**(11), 4636–4636 (2008). 10.1182/blood.V112.11.4636.4636

[225] A. Leftin, N. Ben-Chetrit, J.A. Joyce, J.A. Koutcher, Imaging endogenous macrophage iron deposits reveals a metabolic biomarker of polarized tumor macrophage infiltration and response to CSF1R breast cancer immunotherapy. Sci. Rep. **9**(1), 857 (2019). 10.1038/s41598-018-37408-7 30696910 10.1038/s41598-018-37408-7 PMC6351660

[226] A. Biesemeier, O. Eibl, S. Eswara, J.N. Audinot, T. Wirtz et al., Transition metals and trace elements in the retinal pigment epithelium and choroid: correlative ultrastructural and chemical analysis by analytical electron microscopy and nano-secondary ion mass spectrometry. Metallomics **10**(2), 296–308 (2018). 10.1039/c7mt00259a 29327028 10.1039/c7mt00259a

[227] F. Penen, J. Malherbe, M.P. Isaure, D. Dobritzsch, I. Bertalan et al., Chemical bioimaging for the subcellular localization of trace elements by high contrast TEM, TEM/X-EDS, and NanoSIMS. J. Trace Elem. Med. Biol. **37**, 62–68 (2016). 10.1016/j.jtemb.2016.04.014 27288221 10.1016/j.jtemb.2016.04.014

[228] J. Lovric, N. Najafinobar, M.E. Kurczy, O. De Castro, A. Biesemeier et al., Correlative high-resolution imaging of iron uptake in lung macrophages. Anal. Chem. **94**(37), 12798–12806 (2022). 10.1021/acs.analchem.2c02675 36070604 10.1021/acs.analchem.2c02675 PMC9494303

[229] Y. Zhou, K.T. Que, Z. Zhang, Z.J. Yi, P.X. Zhao et al., Iron overloaded polarizes macrophage to proinflammation phenotype through ROS/acetyl-p53 pathway. Cancer Med. **7**(8), 4012–4022 (2018). 10.1002/cam4.1670 29989329 10.1002/cam4.1670 PMC6089144

[230] H. She, S. Xiong, M. Lin, E. Zandi, C. Giulivi et al., Iron activates NF-kappaB in kupffer cells. Am. J. Physiol. Gastrointest. Liver Physiol. **283**(3), G719-726 (2002). 10.1152/ajpgi.00108.2002 12181188 10.1152/ajpgi.00108.2002

[231] P. Handa, S. Thomas, V. Morgan-Stevenson, B.D. Maliken, E. Gochanour et al., Iron alters macrophage polarization status and leads to steatohepatitis and fibrogenesis. J. Leukoc. Biol. **105**(5), 1015–1026 (2019). 10.1002/JLB.3A0318-108R 30835899 10.1002/JLB.3A0318-108R

[232] H. Kim, J.Y. Hong, W.J. Jeon, J. Lee, Y.J. Lee et al., Melittin regulates iron homeostasis and mediates macrophage polarization in rats with lumbar spinal stenosis. Biomed. Pharmacother. **156**, 113776 (2022). 10.1016/j.biopha.2022.113776 36244265 10.1016/j.biopha.2022.113776

[233] E. Patino, D. Bhatia, S.Z. Vance, A. Antypiuk, R. Uni et al., Iron therapy mitigates chronic kidney disease progression by regulating intracellular iron status of kidney macrophages. JCI Insight **8**(1), e159235 (2023). 10.1172/jci.insight.159235 36394951 10.1172/jci.insight.159235 PMC9870080

[234] D.G. DeNardo, B. Ruffell, Macrophages as regulators of tumour immunity and immunotherapy. Nat. Rev. Immunol. **19**(6), 369–382 (2019). 10.1038/s41577-019-0127-6 30718830 10.1038/s41577-019-0127-6 PMC7339861

[235] H. Zhao, R. Liu, L. Wang, F. Tang, W. Chen et al., Artificial macrophage with hierarchical nanostructure for biomimetic reconstruction of antitumor immunity. Nano-Micro Lett. **15**(1), 216 (2023). 10.1007/s40820-023-01193-4 10.1007/s40820-023-01193-4 PMC10516848 37737506

[236] S. Zanganeh, G. Hutter, R. Spitler, O. Lenkov, M. Mahmoudi et al., Iron oxide nanoparticles inhibit tumour growth by inducing pro-inflammatory macrophage polarization in tumour tissues. Nat. Nanotechnol. **11**(11), 986–994 (2016). 10.1038/nnano.2016.168 27668795 10.1038/nnano.2016.168 PMC5198777

[237] Z. Liu, W. Jiang, J. Nam, J.J. Moon, B.Y.S. Kim, Immunomodulating nanomedicine for cancer therapy. Nano Lett. **18**(11), 6655–6659 (2018). 10.1021/acs.nanolett.8b02340 30185039 10.1021/acs.nanolett.8b02340 PMC6238186

[238] Z. Liu, J. Qiao, T. Nagy, M.P. Xiong, ROS-triggered degradable iron-chelating nanogels: safely improving iron elimination in vivo. J. Control. Release **283**, 84–93 (2018). 10.1016/j.jconrel.2018.05.025 29792889 10.1016/j.jconrel.2018.05.025 PMC6035766

[239] R. Agoro, M. Taleb, V.F.J. Quesniaux, C. Mura, Cell iron status influences macrophage polarization. PLoS ONE **13**(5), e0196921 (2018). 10.1371/journal.pone.0196921 29771935 10.1371/journal.pone.0196921 PMC5957380

[240] H.N. Wilkinson, E.R. Roberts, A.R. Stafford, K.L. Banyard, P. Matteucci et al., Tissue iron promotes wound repair via M2 macrophage polarization and the chemokine (C-C Motif) ligands 17 and 22. Am. J. Pathol. **189**(11), 2196–2208 (2019). 10.1016/j.ajpath.2019.07.01531465751 10.1016/j.ajpath.2019.07.015

[241] Z.S. Gan, Q.Q. Wang, J.H. Li, X.L. Wang, Y.Z. Wang et al., Iron reduces M1 macrophage polarization in RAW264.7 macrophages associated with inhibition of STAT1. Mediators Inflamm. **2017**, 8570818 (2017). 10.1155/2017/8570818 28286378 10.1155/2017/8570818 PMC5327769

[242] D. Li, C. Beisswenger, C. Herr, R.M. Schmid, R.L. Gallo et al., Expression of the antimicrobial peptide cathelicidin in myeloid cells is required for lung tumor growth. Oncogene **33**(21), 2709–2716 (2014). 10.1038/onc.2013.248 23812430 10.1038/onc.2013.248

[243] X. Zhou, W. Yang, J. Li, Ca^2+^- and protein kinase C-dependent signaling pathway for nuclear factor-kappaB activation, inducible nitric-oxide synthase expression, and tumor necrosis factor-alpha production in lipopolysaccharide-stimulated rat peritoneal macrophages. J. Biol. Chem. **281**(42), 31337–31347 (2006). 10.1074/jbc.M602739200 16923814 10.1074/jbc.M602739200

[244] E.Y. Moon, S. Pyo, Lipopolysaccharide stimulates Epac1-mediated Rap1/NF-kappaB pathway in Raw 264.7 murine macrophages. Immunol. Lett. **110**(2), 121–125 (2007). 10.1016/j.imlet.2007.04.002 17532477 10.1016/j.imlet.2007.04.002

[245] L.C. Denlinger, P.L. Fisette, K.A. Garis, G. Kwon, A. Vazquez-Torres et al., Regulation of inducible nitric oxide synthase expression by macrophage purinoreceptors and calcium. J. Biol. Chem. **271**(1), 337–342 (1996). 10.1074/jbc.271.1.337 8550583 10.1074/jbc.271.1.337

[246] Y. Ye, X. Huang, Y. Zhang, X. Lai, X. Wu et al., Calcium influx blocked by SK&K 96365 modulates the LPS plus IFN-gamma-induced inflammatory response in murine peritoneal macrophages. Int. Immunopharmacol. **12**(2), 384–393 (2012). 10.1016/j.intimp.2011.12.011 22212354 10.1016/j.intimp.2011.12.011

[247] S.Y. Ji, H. Lee, H. Hwangbo, S.H. Hong, H.J. Cha et al., A novel peptide oligomer of bacitracin induces M1 macrophage polarization by facilitating Ca^2+^ influx. Nutrients **12**(6), 1603 (2020). 10.3390/nu12061603 32486100 10.3390/nu12061603 PMC7352993

[248] J. Mo, Y. Xu, X. Wang, W. Wei, J. Zhao, Exploiting the protein corona: coating of black phosphorus nanosheets enables macrophage polarization via calcium influx. Nanoscale **12**(3), 1742–1748 (2020). 10.1039/c9nr08570j 31895379 10.1039/c9nr08570j

[249] A. Chauhan, Y. Sun, P. Sukumaran, F.O. Quenum Zangbede, C.N. Jondle et al., M1 macrophage polarization is dependent on TRPC1-mediated calcium entry. iScience **8**, 85–102 (2018). 10.1016/j.isci.2018.09.014 30293012 10.1016/j.isci.2018.09.014 PMC6174824

[250] Z. Yin, T. Ma, Y. Lin, X. Lu, C. Zhang et al., IL-6/STAT3 pathway intermediates M1/M2 macrophage polarization during the development of hepatocellular carcinoma. J. Cell. Biochem. **119**(11), 9419–9432 (2018). 10.1002/jcb.27259 30015355 10.1002/jcb.27259

[251] N. Ding, Y. Wang, C. Dou, F. Liu, G. Guan et al., Physalin D regulates macrophage M1/M2 polarization via the STAT1/6 pathway. J. Cell. Physiol. **234**(6), 8788–8796 (2019). 10.1002/jcp.27537 30317606 10.1002/jcp.27537

[252] H. Jiang, M.B. Harris, P. Rothman, IL-4/IL-13 signaling beyond JAK/STAT. J. Allergy Clin. Immunol. **105**(6 Pt 1), 1063–1070 (2000). 10.1067/mai.2000.107604 10856136 10.1067/mai.2000.107604

[253] G. Nappo, F. Handle, F.R. Santer, R.V. McNeill, R.I. Seed et al., The immunosuppressive cytokine interleukin-4 increases the clonogenic potential of prostate stem-like cells by activation of STAT6 signalling. Oncogenesis **6**(5), e342 (2017). 10.1038/oncsis.2017.23 28553931 10.1038/oncsis.2017.23 PMC5523058

[254] S. Su, Q. Zhao, C. He, D. Huang, J. Liu et al., miR-142-5p and miR-130a-3p are regulated by IL-4 and IL-13 and control profibrogenic macrophage program. Nat. Commun. **6**, 8523 (2015). 10.1038/ncomms9523 26436920 10.1038/ncomms9523 PMC4600756

[255] F.O. Martinez, L. Helming, R. Milde, A. Varin, B.N. Melgert et al., Genetic programs expressed in resting and IL-4 alternatively activated mouse and human macrophages: similarities and differences. Blood **121**(9), e57-69 (2013). 10.1182/blood-2012-06-436212 23293084 10.1182/blood-2012-06-436212

[256] P.J. Murray, J.E. Allen, S.K. Biswas, E.A. Fisher, D.W. Gilroy et al., Macrophage activation and polarization: nomenclature and experimental guidelines. Immunity **41**(1), 14–20 (2014). 10.1016/j.immuni.2014.06.008 25035950 10.1016/j.immuni.2014.06.008 PMC4123412

[257] K. Nelms, A.D. Keegan, J. Zamorano, J.J. Ryan, W.E. Paul, The IL-4 receptor: signaling mechanisms and biologic functions. Annu. Rev. Immunol. **17**, 701–738 (1999). 10.1146/annurev.immunol.17.1.701 10358772 10.1146/annurev.immunol.17.1.701

[258] S.C. Huang, A.M. Smith, B. Everts, M. Colonna, E.L. Pearce et al., Metabolic reprogramming mediated by the mTORC2-IRF4 signaling axis is essential for macrophage alternative activation. Immunity **45**(4), 817–830 (2016). 10.1016/j.immuni.2016.09.016 27760338 10.1016/j.immuni.2016.09.016 PMC5535820

[259] A.S. Divakaruni, W.Y. Hsieh, L. Minarrieta, T.N. Duong, K.K.O. Kim et al., Etomoxir inhibits macrophage polarization by disrupting CoA homeostasis. Cell Metab. **28**(3), 490–50.e37 (2018). 10.1016/j.cmet.2018.06.001 30043752 10.1016/j.cmet.2018.06.001 PMC6125190

[260] J. Lundin, E. Kimby, L. Bergmann, T. Karakas, H. Mellstedt et al., Interleukin 4 therapy for patients with chronic lymphocytic leukaemia: a phase I/II study. Br. J. Haematol. **112**(1), 155–160 (2001). 10.1046/j.1365-2141.2001.02525.x 11167796 10.1046/j.1365-2141.2001.02525.x

[261] Y. Qian, L. Li, Y. Song, L. Dong, P. Chen et al., Surface modification of nanofibrous matrices via layer-by-layer functionalized silk assembly for mitigating the foreign body reaction. Biomaterials **164**, 22–37 (2018). 10.1016/j.biomaterials.2018.02.038 29482061 10.1016/j.biomaterials.2018.02.038

[262] X. Ge, J. Hu, Y. Peng, Z. Zeng, D. He et al., Atmosphere-inspired multilayered nanoarmor with modulable protection and delivery of interleukin-4 for inflammatory microenvironment modulation. Biomaterials **301**, 122254 (2023). 10.1016/j.biomaterials.2023.122254 37531774 10.1016/j.biomaterials.2023.122254

[263] V. Spieler, M.G. Ludwig, J. Dawson, B. Tigani, A. Littlewood-Evans et al., Targeting interleukin-4 to the arthritic joint. J. Control. Release **326**, 172–180 (2020). 10.1016/j.jconrel.2020.07.005 32653504 10.1016/j.jconrel.2020.07.005

[264] R. Lang, D. Patel, J.J. Morris, R.L. Rutschman, P.J. Murray, Shaping gene expression in activated and resting primary macrophages by IL-10. J. Immunol. **169**(5), 2253–2263 (2002). 10.4049/jimmunol.169.5.2253 12193690 10.4049/jimmunol.169.5.2253

[265] P.J. Murray, The primary mechanism of the IL-10-regulated antiinflammatory response is to selectively inhibit transcription. Proc. Natl. Acad. Sci. U.S.A. **102**(24), 8686–8691 (2005). 10.1073/pnas.0500419102 15937121 10.1073/pnas.0500419102 PMC1150817

[266] W. Ouyang, S. Rutz, N.K. Crellin, P.A. Valdez, S.G. Hymowitz, Regulation and functions of the IL-10 family of cytokines in inflammation and disease. Annu. Rev. Immunol. **29**, 71–109 (2011). 10.1146/annurev-immunol-031210-101312 21166540 10.1146/annurev-immunol-031210-101312

[267] J. Patel, IL-10 reprogramming of metabolism in macrophages through mitophagy. Cardiovasc. Res. **113**(11), e40–e41 (2017). 10.1093/cvr/cvx144 28859306 10.1093/cvr/cvx144

[268] K. Minton, Immune regulation: IL-10 targets macrophage metabolism. Nat. Rev. Immunol. **17**(6), 345 (2017). 10.1038/nri.2017.57 28548135 10.1038/nri.2017.57

[269] C.J. Huang, B.R. Stevens, R.B. Nielsen, P.N. Slovin, X. Fang et al., Interleukin-10 inhibition of nitric oxide biosynthesis involves suppression of CAT-2 transcription. Nitric Oxide **6**(1), 79–84 (2002). 10.1006/niox.2001.0402 11829538 10.1006/niox.2001.0402

[270] W.C. Huang, Y.S. Lin, C.Y. Wang, C.C. Tsai, H.C. Tseng et al., Glycogen synthase kinase-3 negatively regulates anti-inflammatory interleukin-10 for lipopolysaccharide-induced iNOS/NO biosynthesis and RANTES production in microglial cells. Immunology **128**(1 Suppl), e275-286 (2009). 10.1111/j.1365-2567.2008.02959.x 19175796 10.1111/j.1365-2567.2008.02959.x PMC2753919

[271] T. Schreiber, S. Ehlers, L. Heitmann, A. Rausch, J. Mages et al., Autocrine IL-10 induces hallmarks of alternative activation in macrophages and suppresses antituberculosis effector mechanisms without compromising T cell immunity. J. Immunol. **183**(2), 1301–1312 (2009). 10.4049/jimmunol.0803567 19561100 10.4049/jimmunol.0803567 PMC2735238

[272] M. Jung, Y. Ma, R.P. Iyer, K.Y. DeLeon-Pennell, A. Yabluchanskiy et al., IL-10 improves cardiac remodeling after myocardial infarction by stimulating M2 macrophage polarization and fibroblast activation. Basic Res. Cardiol. **112**(3), 33 (2017). 10.1007/s00395-017-0622-5 28439731 10.1007/s00395-017-0622-5 PMC5575998

[273] O.P.B. Wiklander, M.A. Brennan, J. Lotvall, X.O. Breakefield, S. El Andaloussi, Advances in therapeutic applications of extracellular vesicles. Sci. Transl. Med. **11**(492), eaav8521 (2019). 10.1126/scitranslmed.aav8521 31092696 10.1126/scitranslmed.aav8521 PMC7104415

[274] D. Li, K. Chen, H. Tang, S. Hu, L. Xin et al., A logic-based diagnostic and therapeutic hydrogel with multi-stimuli responsiveness to orchestrate diabetic bone regeneration. Adv. Mater. **34**(11), e2108430 (2021). 10.1002/adma.202108430 10.1002/adma.202108430 34921569

[275] R. Stienstra, R.T. Netea-Maier, N.P. Riksen, L.A.B. Joosten, M.G. Netea, Specific and complex reprogramming of cellular metabolism in myeloid cells during innate immune responses. Cell Metab. **26**(1), 142–156 (2017). 10.1016/j.cmet.2017.06.001 28683282 10.1016/j.cmet.2017.06.001

[276] M. Munder, K. Eichmann, M. Modolell, Alternative metabolic states in murine macrophages reflected by the nitric oxide synthase/arginase balance: competitive regulation by CD4+ T cells correlates with Th1/Th2 phenotype. J. Immunol. **160**(11), 5347–5354 (1998). 10.4049/jimmunol.160.11.5347 9605134

[277] N. Hannemann, S. Cao, D. Eriksson, A. Schnelzer, J. Jordan et al., Transcription factor Fra-1 targets arginase-1 to enhance macrophage-mediated inflammation in arthritis. J. Clin. Invest. **129**(7), 2669–2684 (2019). 10.1172/JCI96832 30990796 10.1172/JCI96832 PMC6597220

[278] J.K. Dowling, R. Afzal, L.J. Gearing, M.P. Cervantes-Silva, S. Annett et al., Mitochondrial arginase-2 is essential for IL-10 metabolic reprogramming of inflammatory macrophages. Nat. Commun. **12**(1), 1460 (2021). 10.1038/s41467-021-21617-2 33674584 10.1038/s41467-021-21617-2 PMC7936006

[279] C. De Santi, F.K. Nally, R. Afzal, C.P. Duffy, S. Fitzsimons et al., Enhancing arginase 2 expression using target site blockers as a strategy to modulate macrophage phenotype. Mol. Ther. Nucleic Acids **29**, 643–655 (2022). 10.1016/j.omtn.2022.08.004 36090747 10.1016/j.omtn.2022.08.004 PMC9424864

[280] Y. Mao, D. Shi, G. Li, P. Jiang, Citrulline depletion by ASS1 is required for proinflammatory macrophage activation and immune responses. Mol. Cell **82**(3), 527-541.e527 (2022). 10.1016/j.molcel.2021.12.006 35016033 10.1016/j.molcel.2021.12.006

[281] M. Kieler, M. Hofmann, G. Schabbauer, More than just protein building blocks: How amino acids and related metabolic pathways fuel macrophage polarization. FEBS J. **288**(12), 3694–3714 (2021). 10.1111/febs.15715 33460504 10.1111/febs.15715 PMC8359336

[282] W. Ren, Y. Xia, S. Chen, G. Wu, F.W. Bazer et al., Glutamine metabolism in macrophages: a novel target for obesity/type 2 diabetes. Adv. Nutr. **10**(2), 321–330 (2019). 10.1093/advances/nmy084 30753258 10.1093/advances/nmy084 PMC6416106

[283] P.S. Liu, H. Wang, X. Li, T. Chao, T. Teav et al., Alpha-ketoglutarate orchestrates macrophage activation through metabolic and epigenetic reprogramming. Nat. Immunol. **18**(9), 985–994 (2017). 10.1038/ni.3796 28714978 10.1038/ni.3796

[284] M.H. Pai, C.S. Lei, S.T. Su, S.L. Yeh, Y.C. Hou, Effects of dietary glutamine supplementation on immune cell polarization and muscle regeneration in diabetic mice with limb ischemia. Eur. J. Nutr. **59**(3), 921–933 (2020). 10.1007/s00394-019-01951-4 31062080 10.1007/s00394-019-01951-4

[285] E.M. Palmieri, A. Menga, R. Martin-Perez, A. Quinto, C. Riera-Domingo et al., Pharmacologic or genetic targeting of glutamine synthetase skews macrophages toward an M1-like phenotype and inhibits tumor metastasis. Cell Rep. **20**(7), 1654–1666 (2017). 10.1016/j.celrep.2017.07.054 28813676 10.1016/j.celrep.2017.07.054 PMC5575233

[286] X. Shan, P. Hu, L. Ni, L. Shen, Y. Zhang et al., Serine metabolism orchestrates macrophage polarization by regulating the IGF1-p38 axis. Cell. Mol. Immunol. **19**(11), 1263–1278 (2022). 10.1038/s41423-022-00925-7 36180780 10.1038/s41423-022-00925-7 PMC9622887

[287] A.E. Rodriguez, G.S. Ducker, L.K. Billingham, C.A. Martinez, N. Mainolfi et al., Serine metabolism supports macrophage IL-1beta production. Cell Metab. **29**(4), 1003–101.e14 (2019). 10.1016/j.cmet.2019.01.014 30773464 10.1016/j.cmet.2019.01.014 PMC6447453

[288] R. Haas, D. Cucchi, J. Smith, V. Pucino, C.E. Macdougall et al., Intermediates of metabolism: from bystanders to signalling molecules. Trends Biochem. Sci. **41**(5), 460–471 (2016). 10.1016/j.tibs.2016.02.003 26935843 10.1016/j.tibs.2016.02.003

[289] U.E. Martinez-Outschoorn, M. Prisco, A. Ertel, A. Tsirigos, Z. Lin et al., Ketones and lactate increase cancer cell “stemness,” driving recurrence, metastasis and poor clinical outcome in breast cancer: achieving personalized medicine via metabolo-genomics. Cell Cycle **10**(8), 1271–1286 (2011). 10.4161/cc.10.8.15330 21512313 10.4161/cc.10.8.15330 PMC3117136

[290] S.E.A. Selleri, Human mesenchymal stromal cell-secreted lactate induces M2-macrophage differentiation by metabolic reprogramming. Oncotarget **7**(21), 30193–30210 (2016). 10.18632/oncotarget.8623 27070086 10.18632/oncotarget.8623 PMC5058674

[291] R.D. Leone, J.D. Powell, Metabolism of immune cells in cancer. Nat. Rev. Cancer **20**(9), 516–531 (2020). 10.1038/s41568-020-0273-y 32632251 10.1038/s41568-020-0273-y PMC8041116

[292] D. O’Sullivan, D.E. Sanin, E.J. Pearce, E.L. Pearce, Metabolic interventions in the immune response to cancer. Nat. Rev. Immunol. **19**(5), 324–335 (2019). 10.1038/s41577-019-0140-9 30820043 10.1038/s41577-019-0140-9

[293] O.R. Colegio, N.Q. Chu, A.L. Szabo, T. Chu, A.M. Rhebergen et al., Functional polarization of tumour-associated macrophages by tumour-derived lactic acid. Nature **513**(7519), 559–563 (2014). 10.1038/nature13490 25043024 10.1038/nature13490 PMC4301845

[294] D. Zhang, Z. Tang, H. Huang, G. Zhou, C. Cui et al., Metabolic regulation of gene expression by histone lactylation. Nature **574**(7779), 575–580 (2019). 10.1038/s41586-019-1678-1 31645732 10.1038/s41586-019-1678-1 PMC6818755

[295] B. McDonald, A.Z. Zucoloto, I.-L. Yu, R. Burkhard, K. Brown et al., Programing of an intravascular immune firewall by the gut microbiota protects against pathogen dissemination during infection. Cell Host Microbe **28**(5), 660-668.e4 (2020). 10.1016/j.chom.2020.07.014 32810440 10.1016/j.chom.2020.07.014

[296] T. Takeuchi, S. Matile, Sensing applications of synthetic transport systems. Chem. Commun. **49**(1), 19–29 (2013). 10.1039/c2cc36729g 10.1039/c2cc36729g 23154531

[297] H. Wang, C. Wu, X. Tong, S. Chen, A biomimetic metal-organic framework nanosystem modulates immunosuppressive tumor microenvironment metabolism to amplify immunotherapy. J. Control. Release **353**, 727–737 (2023). 10.1016/j.jconrel.2022.11.054 36473607 10.1016/j.jconrel.2022.11.054

[298] Z.X. Liao, Y.C. Fa, I.M. Kempson, S.J. Tseng, Repolarization of M2 to M1 macrophages triggered by lactate oxidase released from methylcellulose hydrogel. Bioconjug. Chem. **30**(10), 2697–2702 (2019). 10.1021/acs.bioconjchem.9b00618 31532192 10.1021/acs.bioconjchem.9b00618

[299] Y.F. Lu, H.S. Li, J. Wang, M.Y. Yao, Y. Peng et al., Engineering bacteria-activated multifunctionalized hydrogel for promoting diabetic wound healing. Adv. Funct. Mater. **31**(48), 210574 (2021). 10.1002/adfm.202105749

[300] E. Blanco, H. Shen, M. Ferrari, Principles of nanoparticle design for overcoming biological barriers to drug delivery. Nat. Biotechnol. **33**(9), 941–951 (2015). 10.1038/nbt.3330 26348965 10.1038/nbt.3330 PMC4978509

[301] Y. Yang, L. Guo, Z. Wang, P. Liu, X. Liu et al., Targeted silver nanoparticles for rheumatoid arthritis therapy via macrophage apoptosis and re-polarization. Biomaterials **264**, 120390 (2021). 10.1016/j.biomaterials.2020.120390 32980634 10.1016/j.biomaterials.2020.120390

[302] F. Wang, L. Wen, J. Liu, W. Peng, Z. Meng et al., Albumin nanocomposites with MnO_2_/Gd_2_O_3_ motifs for precise mr imaging of acute myocardial infarction in rabbit models. Biomaterials **230**, 119614 (2020). 10.1016/j.biomaterials.2019.119614 31753475 10.1016/j.biomaterials.2019.119614

[303] A. Hooftman, C.G. Peace, D.G. Ryan, E.A. Day, M. Yang et al., Macrophage fumarate hydratase restrains mtRNA-mediated interferon production. Nature **615**(7952), 490–498 (2023). 10.1038/s41586-023-05720-6 36890227 10.1038/s41586-019-0000-0 PMC10411300

[304] S. Yeudall, C.M. Upchurch, P.V. Seegren, C.M. Pavelec, J. Greulich et al., Macrophage acetyl-CoA carboxylase regulates acute inflammation through control of glucose and lipid metabolism. Sci. Adv. **8**(47), eadq1984 (2022). 10.1126/sciadv.abq1984 10.1126/sciadv.abq1984 PMC9683712 36417534

[305] J. Ma, K. Wei, J. Liu, K. Tang, H. Zhang et al., Glycogen metabolism regulates macrophage-mediated acute inflammatory responses. Nat. Commun. **11**(1), 1769 (2020). 10.1038/s41467-020-15636-8 32286295 10.1038/s41467-020-15636-8 PMC7156451

[306] Y. Dong, J. Zhang, Y. Wang, Y. Zhang, D. Rappaport et al., Intracavitary spraying of nanoregulator-encased hydrogel modulates cholesterol metabolism of glioma-supportive macrophage for postoperative glioblastoma immunotherapy. Adv. Mater. **36**(13), e2311109 (2024). 10.1002/adma.202311109 38127403 10.1002/adma.202311109

[307] A.C. Thomas, J.T. Mattila, “Of mice and men”: arginine metabolism in macrophages. Front. Immunol. **5**, 479 (2014). 10.3389/fimmu.2014.00479 25339954 10.3389/fimmu.2014.00479 PMC4188127

[308] J.B. Weinberg, M.A. Misukonis, P.J. Shami, S.N. Mason, D.L. Sauls et al., Human mononuclear phagocyte inducible nitric oxide synthase (iNOS): analysis of iNOS mRNA, iNOS protein, biopterin, and nitric oxide production by blood monocytes and peritoneal macrophages. Blood **86**(3), 1184–1195 (1995). 10.1182/blood.V86.3.1184.1184 7542498

[309] S.J. Jackson, G.J. Thomas, Human tissue models in cancer research: Looking beyond the mouse. Dis. Model. Mech. **10**(8), 939–942 (2017). 10.1242/dmm.031260 28768734 10.1242/dmm.031260 PMC5560067

[310] M. Fujii, M. Shimokawa, S. Date, A. Takano, M. Matano et al., A colorectal tumor organoid library demonstrates progressive loss of niche factor requirements during tumorigenesis. Cell Stem Cell **18**(6), 827–838 (2016). 10.1016/j.stem.2016.04.003 27212702 10.1016/j.stem.2016.04.003

[311] D.K. Zhao, J. Liang, X.Y. Huang, S. Shen, J. Wang, Organoids technology for advancing the clinical translation of cancer nanomedicine. Wiley Interdiscip. Rev. Nanomed. Nanobiotechnol. **15**(5), e1892 (2023). 10.1002/wnan.1892 37088100 10.1002/wnan.1892

[312] M. Faria, M. Björnmalm, K.J. Thurecht, S.J. Kent, R.G. Parton et al., Minimum information reporting in bio-nano experimental literature. Nat. Nanotechnol. **13**(9), 777–785 (2018). 10.1038/s41565-018-0246-4 30190620 10.1038/s41565-018-0246-4 PMC6150419

[313] O. Adir, M. Poley, G. Chen, S. Froim, N. Krinsky et al., Integrating artificial intelligence and nanotechnology for precision cancer medicine. Adv. Mater. **32**(13), e1901989 (2020). 10.1002/adma.201901989 31286573 10.1002/adma.201901989 PMC7124889

[314] Y. Wang, S. Sun, Z. Zhang, D. Shi, Nanomaterials for cancer precision medicine. Adv. Mater. **30**(17), e1705660 (2018). 10.1002/adma.201705660 29504159 10.1002/adma.201705660

[315] J.I. Joo, M. Choi, S.H. Jang, S. Choi, S.M. Park et al., Realizing cancer precision medicine by integrating systems biology and nanomaterial engineering. Adv. Mater. **32**(35), e1906783 (2020). 10.1002/adma.201906783 32253807 10.1002/adma.201906783

