# Correction: Nanomaterial-Based Repurposing of Macrophage Metabolism and Its Applications

**DOI:** 10.1007/s40820-024-01491-5

**Published:** 2024-08-14

**Authors:** Tingting Meng, Danfeng He, Zhuolei Han, Rong Shi, Yuhan Wang, Bibo Ren, Cheng Zhang, Zhengwei Mao, Gaoxing Luo, Jun Deng

**Affiliations:** 1 Institute of Burn Research, Southwest Hospital, State Key Laboratory of Trauma and Chemical Poisoning, Army Medical University, Chongqing, 400038 People’s Republic of China; 2 Department of Breast Surgery, Gansu Provincial Hospital, Lanzhou, Gansu 730030 People’s Republic of China; 3 MOE Key Laboratory of Macromolecular Synthesis and Functionalization, Department of Polymer Science and Engineering, Zhejiang University, Hangzhou, 310027 People’s Republic of China

**Correction to: Nano-Micro Letters (2024) 16:246** 10.1007/s40820-024-01455-9

Following publication of the original article [1], the authors reported an error in the last author’s name, it was mistakenly written as “Jun Den”. The correct author’s name “Jun Deng” has been updated in this Correction.

The original article [1] has been corrected.
